# Supplementary material for: What are the research priorities for idiopathic intracranial hypertension? A priority setting partnership between patients and healthcare professionals
Source: BMJ Open. 2019 Mar 15;9(3):e026573. doi: 10.1136/bmjopen-2018-026573 (PMC6429891; doi:10.1136/bmjopen-2018-026573)
Supplement: Supplementary file 1 [file bmjopen-2018-026573supp001.pdf]

Supplemental table 1: The steering group members and their role

Person	Role
Krystal Hemmings	IIH UK Research representative and PSP patient led
Alex Sinclair	Clinical lead and neurologist
Michelle Williamson	IIH UK Chair trustee, project coordinator and carer
Clare Herd	Information specialist
Martin Plowright	IIH patient
Norma-Ann Dan	IIH UK patient representative
Amanda Denton	IIH UK patient representative
Rachel Bennett	IIH patient
Jayne Best	Neuro-Ophthalmologist
Arun Chandran	Neuro-radiologist
Julie Edwards	Headache nurse specialist
Anita Krishnan	Neurologist
Kamal Mahawar	Bariatric surgeon
Susan Mollan	Neuro-Ophthalmologist
Caroline Rick	Trial methodologist
Ahmed Toma	Neurosurgeon
