# Supplementary material for: What are the research priorities for idiopathic intracranial hypertension? A priority setting partnership between patients and healthcare professionals
Source: BMJ Open. 2019 Mar 15;9(3):e026573. doi: 10.1136/bmjopen-2018-026573 (PMC6429891; doi:10.1136/bmjopen-2018-026573)
Supplement: Supplementary file 2 [file bmjopen-2018-026573supp002.pdf]

Supplemental table 2: The prioritisation survey was designed using Qualtrics software (www.qualtrics.com) and responses were requested to the following seven questions:

1. What questions do you have about how the diagnosis of IIH is made?
2. What questions do you have about why people get IIH?
3. What questions do you have about the management of vision in IIH?
4. What questions do you have about the management of headache in IIH?
5. What questions do you have about weight management in IIH?
6. What questions do you have about care provision for patients with IIH? (e.g. General Practice, inpatient, outpatient care)
7. Do you have any other questions about IIH that you feel are important but do not fall into the categories above?
