# Supplementary material for: What are the research priorities for idiopathic intracranial hypertension? A priority setting partnership between patients and healthcare professionals
Source: BMJ Open. 2019 Mar 15;9(3):e026573. doi: 10.1136/bmjopen-2018-026573 (PMC6429891; doi:10.1136/bmjopen-2018-026573)
Supplement: Supplementary file 3 [file bmjopen-2018-026573supp003.pdf]

Supplemental table 3: Partner organisations in alphabetical order

ABN - Association of British Neurologists
BASH - British Association for the Study of Headache
BIOS - British and Irish Orthoptic Society
Fight for Sight – The Eye Research Charity
RCOphth – The Royal College of Ophthalmologists
SBNS CSF subgroup - The Society of British Neurological Surgeons
Shine – Spina bifida, Hydrocephalus, Information, Networking, Equality
The Neurological Alliance
UKNOSIG - The United Kingdom Neuro-Ophthalmology Special Interest Group
