# Supplementary material for: What are the research priorities for idiopathic intracranial hypertension? A priority setting partnership between patients and healthcare professionals
Source: BMJ Open. 2019 Mar 15;9(3):e026573. doi: 10.1136/bmjopen-2018-026573 (PMC6429891; doi:10.1136/bmjopen-2018-026573)
Supplement: Supplementary file 4 [file bmjopen-2018-026573supp004.pdf]

Supplementary Table 4: Characteristics of participants with IIH of first survey

Number	180
Female (%)	96
Median age (years)	35
Ethnicity (%)	
White	92
Black or Asian	3
Multiple ethnic backgrounds	4
Not stated	1
