# Supplementary material for: What are the research priorities for idiopathic intracranial hypertension? A priority setting partnership between patients and healthcare professionals
Source: BMJ Open. 2019 Mar 15;9(3):e026573. doi: 10.1136/bmjopen-2018-026573 (PMC6429891; doi:10.1136/bmjopen-2018-026573)
Supplement: Supplementary file 5 [file bmjopen-2018-026573supp005.pdf]

Supplementary Table 5: Declared specialism of the healthcare professionals in first survey

**Declared specialism of the healthcare professional**	**% of respondents**
Neurologist	45
Ophthalmologist	11
Neurosurgeon	10
Neuro-Ophthalmologist	9
Other	8
Trainee	6
Bariatric Surgeon	3
General Practitioner	3
Nurse	2
Neuroradiologist	1
Orthoptist	1
Not declared	1
