# Supplementary material for: What are the research priorities for idiopathic intracranial hypertension? A priority setting partnership between patients and healthcare professionals
Source: BMJ Open. 2019 Mar 15;9(3):e026573. doi: 10.1136/bmjopen-2018-026573 (PMC6429891; doi:10.1136/bmjopen-2018-026573)
Supplement: Supplementary file 6 [file bmjopen-2018-026573supp006.pdf]

Supplemental table 6: 26 Questions for IIH PSP final workshop in alphabetical order

Are multidisciplinary clinics (joint clinics of neurology, ophthalmology, neurosurgery, dietetics and specialist nurses etc.) clinically and cost effective for the management of IIH and would they improve patient experience?
Are non-invasive intracranial pressure (ICP) measurements accurate and clinically useful?
Can IIH biomarkers (tests in body fluids for example urine, saliva, blood, or brain scans) help diagnosis, predict the risk and guide therapy decisions in IIH?
Can novel therapies for IIH be developed which are effective, safe, and tolerable and potentially help with weight loss as well as reducing brain pressure?
Do lumbar punctures (LPs) have long-term safety complications?
Do the benefits of the drug treatments for IIH outweigh the side effects?
How big is the impact of headache in IIH (how severe are headaches, how often do they occur, how many years do they continue for and how do they impact patients quality of life)?
Is bariatric surgery effective in IIH and at what point in the disease should it be performed?
Is cerebral venous stenosis the cause or consequence of IIH?
Is IIH a lifelong condition?
Is IIH caused by increased production or lack of cerebral spinal fluid (CSF) absorption?
Is there a genetic cause of IIH?
Is there a single or are there multiple causes for IIH?
What are the best ways to monitor visual function?
What are the biological mechanisms of headache in IIH and why in some do headaches continue even after papilloedema has resolved?
What are the hormonal causes for IIH and why is IIH primarily associated with female gender?
What are the triggers for periods of high intracranial pressure (ICP) in people with IIH?
What is happening in the body of a person with IIH which causes the development of the disease, the symptoms and the progression of the disease?
What is the biological explanation for the differences between rapid visual loss compared with gradual visual loss in IIH and how can this be predicted?
What medications are effective and safe to treat IIH headaches?
What other conditions / features are associated with IIH (e.g. depression, sleep apnoea, endocrine disorders, cognition, nerve pain)?
Which is the best type of surgery to treat IIH and when should surgery be performed?
Why do people get IIH without papilloedema (IIHWOP) and how should this be treated?
Why is obesity a risk factor for IIH in women and why is this not the case in men?
With regard to weight loss in IIH: how much is needed to treat IIH and how quickly does it work? What is the best, safest and most acceptable method to achieve this in the short and long term? Additionally, does the initial Body Mass Index (BMI) of the patient have an effect?
Would an education program for health care professionals and patients with IIH improve care and disease experience for IIH patients?
