# Supplementary material for: What are the research priorities for idiopathic intracranial hypertension? A priority setting partnership between patients and healthcare professionals
Source: BMJ Open. 2019 Mar 15;9(3):e026573. doi: 10.1136/bmjopen-2018-026573 (PMC6429891; doi:10.1136/bmjopen-2018-026573)
Supplement: Supplementary file 7 [file bmjopen-2018-026573supp007.pdf]

Supplemental Table 7: Final Top 10 ranked uncertainties for the concerning the treatment and management of people with Idiopathic Intracranial Hypertension

Ranking	Research priority
1	In the individual with IIH; what causes the disease, the symptoms and the progression of the disease?
2	What are the biological mechanisms of headache in IIH and why in some do headaches continue even after papilloedema has resolved?
3	Can new medical therapies for IIH be developed which are effective, safe, and tolerable and potentially help with weight loss as well as reducing brain pressure?
4	What is the biological explanation for the differences between rapid visual loss compared with gradual visual loss in IIH and how can this be predicted?
5	What are the best ways to monitor visual function?
6	Can IIH biomarkers (tests in body fluids for example urine, saliva, blood, or brain scans) help diagnosis, predict the risk and guide therapy decisions in IIH?
7	What are the hormonal causes for IIH and why is IIH primarily associated with female sex?
8	What medications are effective and safe to treat IIH headaches?
9	With regard to weight loss in IIH: how much is needed to treat IIH and how quickly does it work? What is the best, safest and most acceptable method to achieve this in the short and long term? Additionally, does the initial Body Mass Index (BMI) of the patient have an effect?
10	Which is the best type of intervention to treat IIH and when should surgery be performed?
