# Supplementary material for: What are the research priorities for idiopathic intracranial hypertension? A priority setting partnership between patients and healthcare professionals
Source: BMJ Open. 2019 Mar 15;9(3):e026573. doi: 10.1136/bmjopen-2018-026573 (PMC6429891; doi:10.1136/bmjopen-2018-026573)
Supplement: Author's manuscript [file bmjopen-2018-026573.draft_revisions.pdf]

BMJ Open is committed to open peer review. As part of this commitment we make the peer review history of every article we publish publicly available.

When an article is published we post the peer reviewers' comments and the authors' responses online. We also post the versions of the paper that were used during peer review. These are the versions that the peer review comments apply to.

The versions of the paper that follow are the versions that were submitted during the peer review process. They are not the versions of record or the final published versions. They should not be cited or distributed as the published version of this manuscript.

BMJ Open is an open access journal and the full, final, typeset and author-corrected version of record of the manuscript is available on our site with no access controls, subscription charges or pay-per-view fees (http://bmjopen.bmj.com).

If you have any questions on BMJ Open's open peer review process please email editorial.bmjopen@bmj.com

BMJ Open

Top ten research priorities for Idiopathic Intracranial Hypertension (IIH)

Journal:	*BMJ Open*
Manuscript ID	bmjopen-2018-026573
Article Type:	Research
Date Submitted by the Author:	09-Sep-2018
Complete List of Authors:	Mollan, Susan; Institute of Metabolism and Systems Research, College of Medical and Dental Sciences, University of Birmingham; Birmingham Neuro-Ophthalmology Unit, Ophthalmology Department, University Hospitals Birmingham NHS Trust, Queen Elizabeth Hospital Birmingham<br>Hemmings, Krystal; IIH UK<br>Herd, Clare; University of Birmingham, Institute of Applied Health Research<br>Denton, Amanda; IIH UK<br>Williamson, Shelley; Idiopathic Intracranial Hypertension UK,<br>Sinclair, AJ; University of Birmingham, Metabolic Neurology; University Hospitals Birmingham
Keywords:	Idiopathic Intracranial Hypertension, Priority setting, Research priorities, Patient involvement, Service-user involvement, NEUROLOGY

SCHOLARONE™
Manuscripts

For peer review only

Running title: PSP IIH top ten

Top ten research priorities for Idiopathic Intracranial Hypertension (IIH)

Mollan SP* (http://orcid.org/0000-0002-6314-4437),
Hemmings K*,
Herd C,
Denton A,
Williamson M,
Sinclair AJ (orcid.org/0000-0003-2777-5132)

1 Metabolic Neurology, Institute of Metabolism and Systems Research, University of Birmingham, Birmingham, United Kingdom (UK)
2 Birmingham Neuro-Ophthalmology, Queen Elizabeth Hospital, Birmingham, UK
3 IIH UK, Washington, Tyne and Wear, UK
4 Institute of Applied Health Research, University of Birmingham, Edgbaston, UK
5 Department of Neurology, University Hospitals Birmingham, Queen Elizabeth Hospital, Birmingham, UK
6 Centre for Endocrinology, Diabetes and Metabolism, Birmingham Health Partners, Birmingham, UK

* Both authors should be considered joint first author, as they contributed equally to the paper.

Abstract word count: 200
Word count: 1578
References: 13

Corresponding Author: Dr Alexandra J Sinclair, Metabolic Neurology, Institute of Metabolism and Systems Research, University of Birmingham, Birmingham B15 2TT, UK; a.b.sinclair@bham.ac.uk

Key Words: Idiopathic Intracranial Hypertension; Priority setting; Research priorities; Patient involvement; Patient and carer involvement; PPI; Service-user involvement; Impact; Research agenda

For peer review only - http://bmjopen.bmj.com/site/about/guidelines.xhtml

Running title: PSP IIH top ten

Abstract

Objective

Idiopathic Intracranial Hypertension (IIH) is under-researched and the aim was to determine the top 10 research priorities for this disease.

Design

A modified nominal group technique was used to engage participants who had experience of IIH.

Setting

This James Lind Alliance Priority Setting Partnership was commissioned by IIH UK, a charity.

Participants

People with IIH, carers, family and friends, and healthcare professionals participated in two rounds of surveys to identify unique research questions unanswered by current evidence. The most popular 26 uncertainties were presented to stakeholders who then agreed the top 10 topics.

Results

The top 10 research priorities for IIH included aetiology of IIH; the pathological mechanisms of headache in IIH; new treatments in IIH; the difference between acute and gradual visual loss; the best ways to monitor visual function; biomarkers of the disease; hormonal causes of IIH; drug therapies for treatment of headache; weight loss and its role in IIH; and finally, the best intervention to treat IIH and when should surgery be performed.

Conclusions

This priority setting encouraged people with direct experience of IIH to collectively identify critical gaps in the existing evidence. The overarching research aspiration was to understand the aetiology and management of IIH.

For peer review only - http://bmjopen.bmj.com/site/about/guidelines.xhtml

For peer review only

Running title: PSP IIH top ten

Strengths and limitations

- This is the first collaboration of patients, carers and clinicians with experience of Idiopathic Intracranial Hypertension (IIH) to achieve consensus on the priorities for future research.
- The James Lind Alliance (JLA) methods are patient centred and give funding bodies an unbiased agenda for research in IIH.
- Using online surveys as the main method for gathering questions for this Priority Setting Partnership (PSP) may mean that not all those with experience of IIH were aware or able to participate in the process.
- While the JLA process and IIH PSP study recommend those research priorities that are important, there is no guarantee of research funding.

For peer review only

For peer review only - http://bmjopen.bmj.com/site/about/guidelines.xhtml

Running title: PSP IIH top ten

Introduction

Clinical uncertainty in Idiopathic Intracranial Hypertension (IIH) is evident, with the first consensus guidelines for investigation and management stating uncertainties in every aspect of the disease .[1] The 2015 Cochrane review concluded that there is a lack of evidence to guide pharmacological treatment.[2] There are few published randomised clinical trials (RCTs)[3,4] and a small number of ongoing trials.[5,6] Research is infrequent due to the rarity of the IIH [7,8] and the lack of understanding of the underlying pathology.[9]

IIH predominantly affects overweight women of childbearing age with the incidence of the disease predicted to rise with the increasing global prevalence of obesity.[7,8] In those with severely affected vision, surgery may be indicated.[1] For the majority, it can be a chronic condition, with headaches impacting on the quality of life of patients, [10] and an economic burden [11].

Understanding where research should be directed was a priority for IIH UK, the leading charity for IIH in the United Kingdom (UK). The James Lind Alliance (JLA), a UK National Institute for Health Research-supported initiative, aims to provide a transparent process that enables patients and healthcare professionals (HCP) to work together to agree on the most important uncertainties to inform the research agenda. The aim of this IIH priority setting partnership (PSP), was to identify gaps in knowledge that matter most to key stakeholders (patients, carers and clinicians), and to indicate where future funding should be placed.

For peer review only - http://bmjopen.bmj.com/site/about/guidelines.xhtml

Running title: PSP IIH top ten

Methods

Patient and public involvement

This research priority partnership was established by IIH UK, a charity that is run by carers and people with IIH. At each stage of the JLA process, patients and carers were equal collaborators in the design and decisions including the survey design and piloting, survey participation and the final workshop. They disseminated the surveys on the charity website and via social media. All participants were able to indicate a desire for further involvement and for information about the results.

IIH PSP process

The University of Birmingham, UK, acted as academic partner to the IIH PSP and the process was led by the IIH UK research representative, in collaboration with the James Lind Alliance (JLA, www.jla.nihr.ac.uk). A steering group with representation from IIH UK, patients and all the major specialities associated with IIH plus an independent information specialist oversaw the process (supplemental table 1). In February 2017, key organisations accepted the invitation to become partners. They included the Association of British Neurologists; the British Association for the Study of Headache; the British and Irish Orthoptic Society; Fight for Sight; The Royal College of Ophthalmologists; The Society of British Neurological Surgeons CSF group; Shine; Neurological Alliance and the United Kingdom Neuro-Ophthalmology Special Interest Group (supplemental table 2). The PSP stages were broadly based on the four step process developed by the JLA (figure 1).[12]

This PSP was concerned with adult IIH only and any responses relating to children were excluded. Responses concerning the classification of the disease, healthcare funding/entitlements, or statements without a discernible question were excluded.

For peer review only - http://bmjopen.bmj.com/site/about/guidelines.xhtml

The IIH UK internal review board formally reviewed the project and further ethical approval was not required. All data was anonymised and sent to the information specialist at the University of Birmingham for processing.

The prioritisation survey questions were constructed (supplemental table 3) and the survey was advertised by partners (supplemental table 2), IIH UK and steering group members. All responses were refined to understandable 'uncertainties' with the exception of those considered to be 'out of scope'. These were categorised using the UK Clinical Research Collaboration Health Research Classification System, sorted into themes and then formulated into indicative questions by steering group members. A literature search was conducted with the electronic databases CENTRAL, Embase and Medline searched from inception to March 2018 for systematic reviews using strategies based on those used by Piper *at al*. [2]. The "known knowns" with reference to the appropriate literature and duplicate questions were removed. Questions were amalgamated when practical to do so. The long list was then verified by the PSP lead and discussions were held with the wider steering group if disagreements occurred.

The known unknowns were then used for the interim survey. Respondents ranked the questions, returning their top ten. The rankings were reverse scored and the total scores for the two groups: individuals with IIH, friends, or carers; and HCP, were calculated separately to ensure an equal weighting. The most popular 26 questions were then taken forward, which included the top 10 for both groups, to the final workshop, with the aim of consensus on the top 10 priorities.[12]

For peer review only - http://bmjopen.bmj.com/site/about/guidelines.xhtml

Results

The prioritisation survey generated 356 responses (figure 1). Demographic data for those with IIH is provided in supplemental table 5 and details of HCP specialisms in supplemental table 6. Of the 2,405 generated uncertainties, 140 were out of scope. The resulting 2,265 were grouped into 64 indicative questions. Sixteen were deemed to be already known or unanswerable by research, leaving 48 questions for presentation in the interim survey. Responses from 512 people were collected in a ratio of 4:1 people with IIH, friends and carers to HCP.

A final list of 26 prioritised questions was generated from the analysis of the interim survey, which included the top 10 for both groups (supplemental table 4). The commonest themes from non-HPC was why the disease develops and progresses; hormonal causes and female predominance; and the conditions associated with IIH. For HCP education; the utility of biomarkers; and biological mechanisms of headache were the commonest. At the consensus workshop the top 10 priorities were agreed (figure 2; supplemental table 7).

For peer review only - http://bmjopen.bmj.com/site/about/guidelines.xhtml

Discussion

Understanding the most relevant research projects to fund can be challenging. It is imperative that the topics identified in a disease area have the utmost relevance to patients affected by the disease and recognised by clinicians that have clear understanding of the clinical entity. We have undertaken a JLA PSP to establish the top 10 research areas for IIH.

The IIH JLA PSP was funded by IIH UK and set up those who have an active collaboration to improve care for people with IIH.[13] The principles and structured process outlined by the JLA was adhered to steadfastly throughout.[12] All data was maintained in a manner that could be tracked back at any point to the original questions and demographic source; this provided transparency.

Within the feedback people with IIH voiced that they felt their opinions were often not heard. There was a good response rate from all groups when considering how rare IIH is. Submissions with low duplication rates were not removed, a process which can introduce bias. All submitted uncertainties were considered in the long list if they were determined to be known unknowns, including those asked by a single respondent. The data analysis followed standard protocols, though it was complicated by the use of multiple questions in the initial survey (supplemental table 3) as each respondent could appear in up to seven separate initial categories.

For peer review only - http://bmjopen.bmj.com/site/about/guidelines.xhtml

For peer review only

Despite the use of identification codes, the multi-level process meant that the number of individuals contributing to the final data set could not be reasonably calculated. The project took 18 months and surveys were closed on schedule, leaving the possibility that this happended before the maximum number of respondents could contribute.

A major challenge for the IIH PSP steering group was to engage all the relevant HCP (namely neurologists, ophthalmologists, neurosurgeons, radiologists, orthoptists). The speciality diversity brought strength to the process and allowed for a broad inclusion, however during the final selection for the top ten, clinicians were clearly polarised by their individual specialism. For example, surgeons were keen for novel interventions, whereas physicians were promoting better medical therapies.

At the interim survey it was clear there was a discrepancy between the non-HCP and HCP in their most popular themes, with patients keen for research into the aetiology, and HCP more commonly ranked education, biomarkers and the pathological mechanisms driving headache. The top priority of the patients' group at the interim survey, was the same as the final result of the consensus workshop.

Some differing opinions between non-HCP and HCP were expressed at the workshop. One issue was surrounding weight loss, seen by physicians as the only disease modifiable therapy and so a high priority for further understanding. This was a highly sensitive issue among the patients and carers present who voiced that it was not considered so important

For peer review only - http://bmjopen.bmj.com/site/about/guidelines.xhtml

by patients. During the workshop a collective decision was made to have a wide scope within the top 10 areas. If a topic was already featured high within the list, questions that contained a similar theme were purposely voted lower. For example, weight loss, the longer more detailed question was ranked higher than the question regarding bariatric surgery, with the reasoning that it could be answered not only by the weight loss question but also by number 10: the intervention question. For this reason, no further ranking below the top 10 should be published. Of note two areas that did not feature in the top 10, namely multidisciplinary clinics and an education program. They were scored as important during the interim survey, particularly by HPC. The consensus workshop delegates agreed that although these are highly important, the PSP is intended to inform grant bodies who fund research and these areas were universally accepted to require improvement.

The IIH PSP has been an opportunity to understand the areas that are important to all. It has the potential to influence the research agenda and consequently treatment and management of this idiopathic disease.

For peer review only

For peer review only - http://bmjopen.bmj.com/site/about/guidelines.xhtml

References

1. Mollan, S.P., et al., *Idiopathic intracranial hypertension: consensus guidelines on management.* J Neurol Neurosurg Psychiatry, 2018.
2. Piper, R.J., et al., *Interventions for idiopathic intracranial hypertension.* Cochrane Database Syst Rev, 2015(8): p. CD003434.
3. Ball, A.K., et al., *A randomised controlled trial of treatment for idiopathic intracranial hypertension.* J Neurol, 2011. **258**(5): p. 874-81.
4. Committee, N.I.I.H.S.G.W., et al., *Effect of acetazolamide on visual function in patients with idiopathic intracranial hypertension and mild visual loss: the idiopathic intracranial hypertension treatment trial.* JAMA, 2014. **311**(16): p. 1641-51.
5. Markey, K.A., et al., *Assessing the Efficacy and Safety of an 11β-Hydroxysteroid Dehydrogenase Type 1 Inhibitor (AZD4017) in the Idiopathic Intracranial Hypertension Drug Trial, IIH:DT: Clinical Methods and Design for a Phase II Randomized Controlled Trial.* JMIR Research Protocols, 2017. **6**(9): p. e181.
6. Ottridge, R., et al., *Randomised controlled trial of bariatric surgery versus a community weight loss programme for the sustained treatment of idiopathic intracranial hypertension: the Idiopathic Intracranial Hypertension Weight Trial (IIH:WT) protocol.* BMJ Open, 2017. **7**(9).
7. Markey, K.A., et al., *Understanding idiopathic intracranial hypertension: mechanisms, management, and future directions.* Lancet Neurol, 2016. **15**(1): p. 78-91.
8. Mollan, S.P., et al., *Evolving evidence in adult idiopathic intracranial hypertension: pathophysiology and management.* J Neurol Neurosurg Psychiatry, 2016. **87**(9): p. 982-92.
9. Hornby, C., et al., *Metabolic Concepts in Idiopathic Intracranial Hypertension and Their Potential for Therapeutic Intervention.* Journal of neuro-ophthalmology : the official journal of the North American Neuro-Ophthalmology Society, 2018.
10. Mulla Y, Markey KA, Woolley RL, Patel S, Mollan SP, Sinclair AJ. Headache determines quality of life in idiopathic intracranial hypertension. *The Journal of Headache and Pain*. 2015;16:45. doi:10.1186/s10194-015-0521-9.
11. Friesner D, Rosenman R, Lobb BM, Tanne, E. Idiopathic intracranial hypertension in the USA: the role of obesity in establishing prevalence and healthcare costs. Obesity Reviews 2011; 12: e372–e380
12. Cowan, K. and S. Oliver, *The James Lind Alliance Guidebook*. 2016: http://www.jla.nihr.ac.uk/jla-guidebook/.
13. Scotton WJ, Mollan SP, Walters T, et al. Characterising the patient experience of diagnostic lumbar puncture in idiopathic intracranial hypertension: a cross-sectional online survey. *BMJ Open*. 2018;8(5):e020445. doi:10.1136/bmjopen-2017-020445.

For peer review only - http://bmjopen.bmj.com/site/about/guidelines.xhtml

Acknowledgements:

We would like to acknowledge all the people who contributed to the surveys. We would also like to acknowledge the support from the partner organisations: Association of British Neurologists; British Association for the Study of Headache; British and Irish Orthoptic Society; Fight for Sight – The Eye Research Charity; The Royal College of Ophthalmologists; The Society of British Neurological Surgeons CSF group; Shine; The Neurological Alliance and The United Kingdom Neuro-Ophthalmology Special Interest Group.

Declarations or Conflicts of Interest

No authors contributing have a conflict of interest in the subject matter.

AJS is funded by an NIHR Clinician Scientist Fellowship (NIHR-CS-011-028) and by the Medical Research Council, UK (MR/K015184/1).

Contributor Statement

Mollan SP: interpretation of the survey results; drafting and review of the manuscript.
Hemmings K: PSP patient lead; administration of both surveys; drafting and review of the manuscript.
Herd C: literature review; independent information specialist; drafting and review of the manuscript.
Denton A**:** critical review of the manuscript.
Williamson M: organisation of the consensus final workshop; and critical review of the manuscript.
Sinclair AJ: PSP clinical lead; interpretation of the survey results and critical review of the manuscript.

All authors were steering group members and have read and approved the final manuscript.

Legend to figures:

Figure 1: Consort diagram and details of the JLA IIH PSP
Figure 2: Final Top 10 ranked uncertainties for the concerning the treatment and management of people with Idiopathic Intracranial Hypertension

For peer review only - http://bmjopen.bmj.com/site/about/guidelines.xhtml

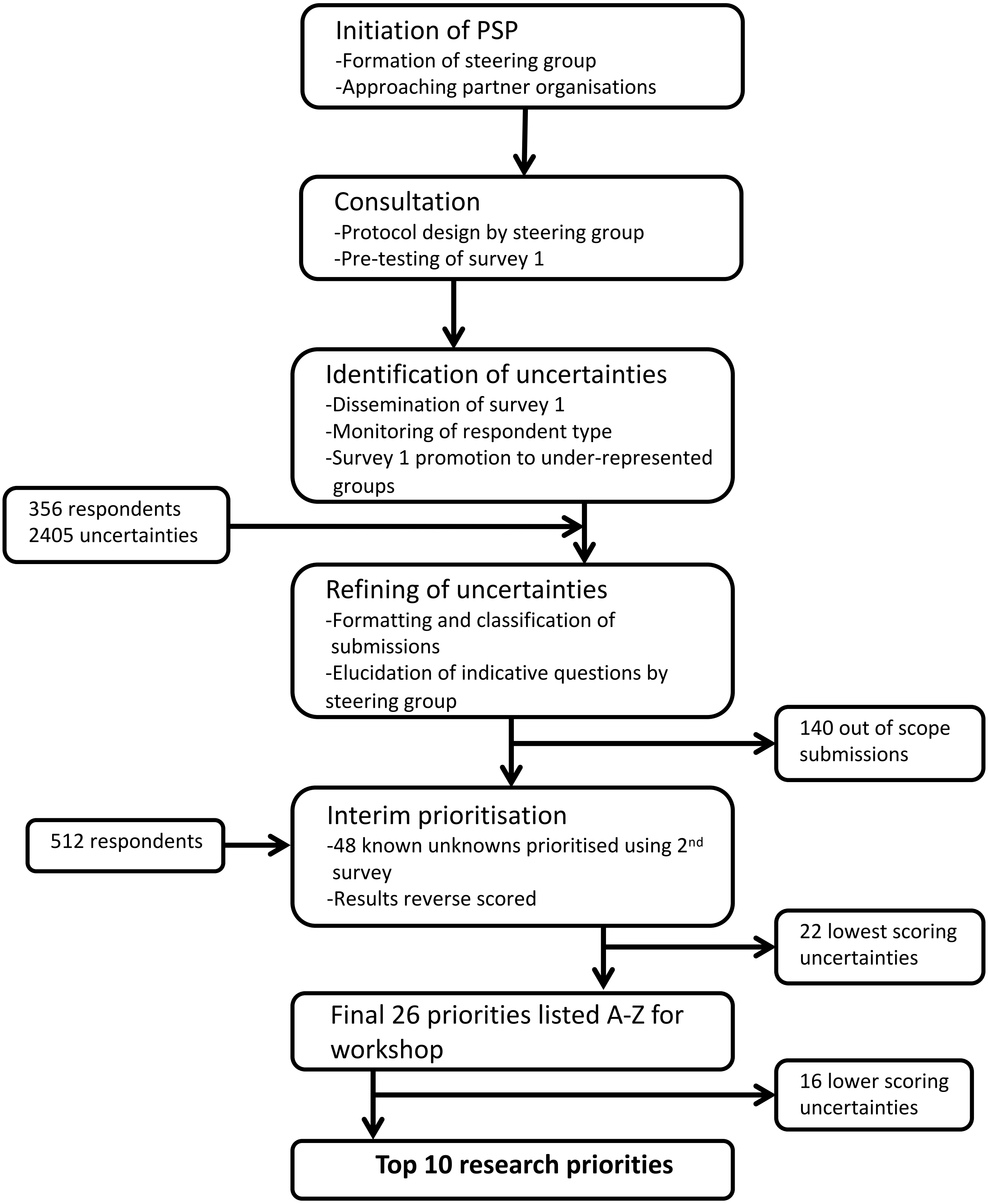
Initiation of PSP
-Formation of steering group
-Approaching partner organisations
Consultation
-Protocol design by steering group
-Pre-testing of survey 1
Identification of uncertainties
-Dissemination of survey 1
-Monitoring of respondent type
-Survey 1 promotion to under-represented groups
356 respondents
2405 uncertainties
Refining of uncertainties
-Formatting and classification of submissions
-Elucidation of indicative questions by steering group
140 out of scope submissions
512 respondents
Interim prioritisation
-48 known unknowns prioritised using 2nd survey
-Results reverse scored
22 lowest scoring uncertainties
Final 26 priorities listed A-Z for workshop
16 lower scoring uncertainties
Top 10 research priorities

IDIOPATHIC INTRACRANIAL HYPERTENSION PRIORITY SETTING PARTNERSHIP

Top 10 Priorities for IIH Research

1 CAUSES OF IIH

In the individual with IIH; what causes the disease, the symptoms and the progression of the disease?

2 MECHANISMS OF HEADACHE

What are the biological mechanisms of headache in IIH and why in some do headaches continue even after papilloedema has resolved?

3 NEW THERAPIES FOR IIH

Can new medical therapies for IIH be developed which are effective, safe, and tolerable and potentially help with weight loss as well as reducing brain

4 PREDICTING VISUAL LOSS

What is the biological explanation for the differences between rapid visual loss compared with gradual visual loss in IIH and how can this be predicted?

5 MONITORING VISUAL FUNCTION

What are the best ways to monitor visual function?

6 IIH BIOMARKERS

Can IIH biomarkers (tests in body fluids for example urine, saliva, blood, or brain scans) help diagnosis, predict the risk and guide therapy decisions in IIH?

7 HORMONES

What are the hormonal causes for IIH and why is IIH primarily associated with female sex?

8 MEDICATION TO TREAT HEADACHE

What medications are effective and safe to treat IIH headaches?

9 WEIGHT LOSS

With regard to weight loss in IIH: how much is needed to treat IIH and how quickly does it work? What is the best, safest and most acceptable method to achieve this in the short and long term? Additionally, does the initial Body Mass Index (BMI) of the patient have an effect?

10 BEST TYPE OF INTERVENTION

Which is the best type of intervention to treat IIH and when should surgery be performed?

IIHUK Idiopathic Intracranial Hypertension

James Lind Alliance Priority Setting Partnerships

IIH PATIENTS AND PROFESSIONALS SETTING RESEARCH PRIORITIES

For peer review only

For peer review only - http://bmjopen.bmj.com/site/about/guidelines.xhtml

Supplemental table 1: The steering group members and their role

Person	Role
Krystal Hemmings	IIH UK Research representative and PSP patient led
Alex Sinclair	Clinical lead and neurologist
Michelle Williamson	IIH UK Chair trustee, project coordinator and carer
Clare Herd	Information specialist
Martin Plowright	IIH patient
Norma-Ann Dan	IIH UK patient representative
Amanda Denton	IIH UK patient representative
Rachel Bennett	IIH patient
Jayne Best	Neuro-Ophthalmologist
Arun Chandran	Neuro-radiologist
Julie Edwards	Headache nurse specialist
Anita Krishnan	Neurologist
Kamal Mahawar	Bariatric surgeon
Susan Mollan	Neuro-Ophthalmologist
Caroline Rick	Trial methodologist
Ahmed Toma	Neurosurgeon

For peer review only

Supplemental table 2: Partner organisations in alphabetical order

ABN - Association of British Neurologists
BASH - British Association for the Study of Headache
BIOS - British and Irish Orthoptic Society
Fight for Sight – The Eye Research Charity
RCOphth – The Royal College of Ophthalmologists
SBNS CSF subgroup - The Society of British Neurological Surgeons
Shine – Spina bifida, Hydrocephalus, Information, Networking, Equality
The Neurological Alliance
UKNOSIG - The United Kingdom Neuro-Ophthalmology Special Interest Group

For peer review only

Supplemental table 3: The prioritisation survey was designed using Qualtrics software (www.qualtrics.com) and responses were requested to the following seven questions:

1. What questions do you have about how the diagnosis of IIH is made?
2. What questions do you have about why people get IIH?
3. What questions do you have about the management of vision in IIH?
4. What questions do you have about the management of headache in IIH?
5. What questions do you have about weight management in IIH?
6. What questions do you have about care provision for patients with IIH? (e.g. General Practice, inpatient, outpatient care)
7. Do you have any other questions about IIH that you feel are important but do not fall into the categories above?

For peer review only

Supplemental table 4: 26 Questions for IIH PSP final workshop in alphabetical order

Are multidisciplinary clinics (joint clinics of neurology, ophthalmology, neurosurgery, dietetics and specialist nurses etc.) clinically and cost effective for the management of IIH and would they improve patient experience?
Are non-invasive intracranial pressure (ICP) measurements accurate and clinically useful?
Can IIH biomarkers (tests in body fluids for example urine, saliva, blood, or brain scans) help diagnosis, predict the risk and guide therapy decisions in IIH?
Can novel therapies for IIH be developed which are effective, safe, and tolerable and potentially help with weight loss as well as reducing brain pressure?
Do lumbar punctures (LPs) have long-term safety complications?
Do the benefits of the drug treatments for IIH outweigh the side effects?
How big is the impact of headache in IIH (how severe are headaches, how often do they<br>occur, how many years do they continue for and how do they impact patients quality of life)?
Is bariatric surgery effective in IIH and at what point in the disease should it be performed?
Is cerebral venous stenosis the cause or consequence of IIH?
Is IIH a lifelong condition?
Is IIH caused by increased production or lack of cerebral spinal fluid (CSF) absorption?
Is there a genetic cause of IIH?
Is there a single or are there multiple causes for IIH?
What are the best ways to monitor visual function?
What are the biological mechanisms of headache in IIH and why in some do headaches<br>continue even after papilloedema has resolved?
What are the hormonal causes for IIH and why is IIH primarily associated with female gender?
What are the triggers for periods of high intracranial pressure (ICP) in people with IIH?
What is happening in the body of a person with IIH which causes the development of the disease, the symptoms and the progression of the disease?
What is the biological explanation for the differences between rapid visual loss compared with gradual visual loss in IIH and how can this be predicted?
What medications are effective and safe to treat IIH headaches?
What other conditions / features are associated with IIH (e.g. depression, sleep apnoea, endocrine disorders, cognition, nerve pain)?
Which is the best type of surgery to treat IIH and when should surgery be performed?
Why do people get IIH without papilloedema (IIHWOP) and how should this be treated?
Why is obesity a risk factor for IIH in women and why is this not the case in men?
With regard to weight loss in IIH: how much is needed to treat IIH and how quickly does it work? What is the best, safest and most acceptable method to achieve this in the short and long term? Additionally, does the initial Body Mass Index (BMI) of the patient have an effect?

For peer review only

Would an education program for health care professionals and patients with IIH improve care and disease experience for IIH patients?

For peer review only

Supplementary Table 5: Characteristics of participants with IIH of first survey

Number	180
Female (%)	96
Median age (years)	35
Ethnicity (%)	
White	92
Black or Asian	3
Multiple ethnic backgrounds	4
Not stated	1

For peer review only

Supplementary Table 6: Declared specialism of the healthcare professionals in first survey

Declared specialism of the healthcare professional	% of respondents
Neurologist	45
Ophthalmologist	11
Neurosurgeon	10
Neuro-Ophthalmologist	9
Other	8
Trainee	6
Bariatric Surgeon	3
General Practitioner	3
Nurse	2
Neuroradiologist	1
Orthoptist	1
Not declared	1

For peer review only

Supplemental Table 7: Final Top 10 ranked uncertainties for the concerning the treatment and management of people with Idiopathic Intracranial Hypertension

Ranking	Research priority
1	In the individual with IIH; what causes the disease, the symptoms and the progression of the disease?
2	What are the biological mechanisms of headache in IIH and why in some do headaches continue even after papilloedema has resolved?
3	Can new medical therapies for IIH be developed which are effective, safe, and tolerable and potentially help with weight loss as well as reducing brain pressure?
4	What is the biological explanation for the differences between rapid visual loss compared with gradual visual loss in IIH and how can this be predicted?
5	What are the best ways to monitor visual function?
6	Can IIH biomarkers (tests in body fluids for example urine, saliva, blood, or brain scans) help diagnosis, predict the risk and guide therapy decisions in IIH?
7	What are the hormonal causes for IIH and why is IIH primarily associated with female sex?
8	What medications are effective and safe to treat IIH headaches?
9	With regard to weight loss in IIH: how much is needed to treat IIH and how quickly does it work? What is the best, safest and most acceptable method to achieve this in the short and long term? Additionally, does the initial Body Mass Index (BMI) of the patient have an effect?
10	Which is the best type of intervention to treat IIH and when should surgery be performed?

For peer review only

BMJ Open

Research priorities for idiopathic intracranial hypertension

Journal:	*BMJ Open*
Manuscript ID	bmjopen-2018-026573.R1
Article Type:	Research
Date Submitted by the Author:	16-Dec-2018
Complete List of Authors:	Mollan, Susan; University Hospitals Birmingham NHS Foundation Trust<br>Hemmings, Krystal; IIH UK<br>Herd, Clare; University of Birmingham, Institute of Applied Health Research<br>Denton, Amanda; IIH UK<br>Williamson, Shelley; Idiopathic Intracranial Hypertension UK,<br>Sinclair, AJ; University of Birmingham, Metabolic Neurology; University Hospitals Birmingham
**Primary Subject Heading**:	Neurology
Secondary Subject Heading:	Ophthalmology, Research methods, Patient-centred medicine
Keywords:	Idiopathic Intracranial Hypertension, Priority setting, Research priorities, Patient involvement, Service-user involvement, NEUROLOGY

SCHOLARONE™
Manuscripts

Running title: PSP IIH top ten

Research priorities for idiopathic intracranial hypertension

Mollan SP* (http://orcid.org/0000-0002-6314-4437) [1]
Hemmings K* [2]
Herd C [3]
Denton A [2]
Williamson S [2]
Sinclair AJ (orcid.org/0000-0003-2777-5132)[4,5,6]

1 Birmingham Neuro-Ophthalmology, Queen Elizabeth Hospital, Birmingham, UK
2 IIH UK, Washington, Tyne and Wear, UK
3 Institute of Applied Health Research, University of Birmingham, Edgbaston, UK
4 Metabolic Neurology, Institute of Metabolism and Systems Research, University of Birmingham, Birmingham, United Kingdom (UK)
5 Department of Neurology, University Hospitals Birmingham, Queen Elizabeth Hospital, Birmingham, UK
6 Centre for Endocrinology, Diabetes and Metabolism, Birmingham Health Partners, Birmingham, UK

* Both authors should be considered joint first author, as they contributed equally to the paper.

Abstract word count: 200
Word count: 1873
References: 19

Corresponding Author: Dr Alexandra J Sinclair, Metabolic Neurology, Institute of Metabolism and Systems Research, University of Birmingham, Birmingham B15 2TT, UK; a.b.sinclair@bham.ac.uk

Key Words: Idiopathic Intracranial Hypertension; Priority setting; Research priorities; Patient involvement; Patient and carer involvement; PPI; Service-user involvement; Impact; Research agenda

For peer review only - http://bmjopen.bmj.com/site/about/guidelines.xhtml

Running title: PSP IIH top ten

Abstract

Objective

Idiopathic Intracranial Hypertension (IIH) is under-researched and the aim was to determine the top 10 research priorities for this disease.

Design

A modified nominal group technique was used to engage participants who had experience of IIH.

Setting

This James Lind Alliance Priority Setting Partnership was commissioned by IIH UK, a charity.

Participants

People with IIH, carers, family and friends, and healthcare professionals participated in two rounds of surveys to identify unique research questions unanswered by current evidence. The most popular 26 uncertainties were presented to stakeholders who then agreed the top 10 topics.

Results

The top 10 research priorities for IIH included aetiology of IIH; the pathological mechanisms of headache in IIH; new treatments in IIH; the difference between acute and gradual visual loss; the best ways to monitor visual function; biomarkers of the disease; hormonal causes of IIH; drug therapies for treatment of headache; weight loss and its role in IIH; and finally, the best intervention to treat IIH and when should surgery be performed.

Conclusions

This priority setting encouraged people with direct experience of IIH to collectively identify critical gaps in the existing evidence. The overarching research aspiration was to understand the aetiology and management of IIH.

For peer review only - http://bmjopen.bmj.com/site/about/guidelines.xhtml

Running title: PSP IIH top ten

Strengths and limitations

- This is the first collaboration of patients, carers and clinicians with experience of Idiopathic Intracranial Hypertension (IIH) to achieve consensus on the priorities for future research.
- The James Lind Alliance (JLA) methods are patient centred and give funding bodies an unbiased agenda for research in IIH.
- Using online surveys as the main method for gathering questions for this Priority Setting Partnership (PSP) may mean that not all those with experience of IIH were aware or able to participate in the process.
- It is conceivable that possibly all the research questions gathered are not exhaustive.
- While the JLA process and IIH PSP study recommend those research priorities that are important, there is no guarantee of research funding.

For peer review only

For peer review only - http://bmjopen.bmj.com/site/about/guidelines.xhtml

Running title: PSP IIH top ten

Introduction

Clinical uncertainty in Idiopathic Intracranial Hypertension (IIH) is evident, with the first consensus guidelines for investigation and management stating uncertainties in every aspect of the disease .[1] The 2015 Cochrane review concluded that there is a lack of evidence to guide pharmacological treatment.[2] There are few published randomised clinical trials (RCTs)[3,4] and a small number of ongoing trials.[5,6] Research is infrequent due to the rarity of the IIH [7,8] and the lack of understanding of the underlying pathology.[9]

IIH predominantly affects overweight women of childbearing age with the incidence of the disease documented to be rising [10] with the increasing prevalence of obesity.[7,8] In those with severely affected vision, surgery may be indicated.[1] For the majority, it can be a chronic condition, with headaches impacting on the quality of life of patients, [11] and an economic burden [10,12].

Understanding where research should be directed was a priority for IIH UK, the leading charity for IIH in the United Kingdom (UK). The James Lind Alliance (JLA), a UK National Institute for Health Research-supported initiative, aims to provide a transparent process that enables patients and healthcare professionals (HCP) to work together to agree on the most important uncertainties to inform the research agenda. The aim of this IIH priority setting partnership (PSP), was to identify gaps in knowledge that matter most to key stakeholders (patients, carers and clinicians), and to indicate where future funding should be placed.

For peer review only - http://bmjopen.bmj.com/site/about/guidelines.xhtml

Running title: PSP IIH top ten

Methods

Patient and public involvement

This research priority partnership was established by IIH UK, a charity that is run by carers and people with IIH. At each stage of the JLA process, patients and carers were equal collaborators in the design and decisions including the survey design and piloting, survey participation and the final workshop. They disseminated the surveys on the charity website and via social media. All participants were able to indicate a desire for further involvement and for information about the results.

IIH PSP process

The University of Birmingham, UK, acted as academic partner to the IIH PSP and the process was led by the IIH UK research representative, in collaboration with the James Lind Alliance (JLA, www.jla.nihr.ac.uk). A steering group with representation from IIH UK, patients and all the major specialities associated with IIH plus an independent information specialist oversaw the process (supplemental table 1). In February 2017, key organisations accepted the invitation to become partners. They included the Association of British Neurologists; the British Association for the Study of Headache; the British and Irish Orthoptic Society; Fight for Sight; The Royal College of Ophthalmologists; The Society of British Neurological Surgeons CSF group; Shine; Neurological Alliance and the United Kingdom Neuro-Ophthalmology Special Interest Group (supplemental table 2). The PSP stages were broadly based on the four step process developed by the JLA (figure 1).[13]

This PSP was concerned with adult IIH only and any responses exclusively relating to children were excluded. There was limited funding for the project, and including the paediatric population would have required funding for two different work streams. It is well

For peer review only - http://bmjopen.bmj.com/site/about/guidelines.xhtml

documented the expectantly different phenotype between adult and those prepubescent children with IIH.[14] However responses were not limited by those who submitted, and hence those with children with IIH are likely to be included. Indeed, at the final stakeholder meeting there was representation from carers of children with IIH. Responses concerning the classification of the disease, healthcare funding/entitlements, or statements without a discernible question were excluded.

The IIH UK internal review board formally reviewed the project and further ethical approval was not required. All data was anonymised and sent to the information specialist at the University of Birmingham for processing.

The prioritisation survey questions were constructed (supplemental table 2) by the steering group, aided by the first guidelines in IIH where uncertainties exist around the diagnosis, investigation and management.[1] This first survey was advertised by partners (supplemental table 3), IIH UK and steering group members. All responses were refined to understandable 'uncertainties' with the exception of those considered to be 'out of scope'. These were categorised using the UK Clinical Research Collaboration Health Research Classification System, sorted into themes and then formulated into indicative questions by steering group members, working in groups with at least one HCP and one patient representative. A literature search was conducted with the electronic databases CENTRAL, Embase and Medline searched from inception to March 2018 for systematic reviews using strategies based on those used by Piper *at al*. [2]. The "known knowns" with reference to the appropriate literature and duplicate questions were removed. Questions were amalgamated when

For peer review only - http://bmjopen.bmj.com/site/about/guidelines.xhtml

practical to do so. The long list was then verified by the PSP lead and discussions were held with the wider steering group if disagreements occurred.

The known unknowns were then used for the interim survey. Respondents ranked the questions, returning their top ten. The rankings were reverse scored and the total scores for the two groups: individuals with IIH, friends, or carers; and HCP, were calculated separately to ensure an equal weighting. The most popular 26 questions were then taken forward, which included the top 10 for both groups, to the final workshop, with the aim of consensus on the top 10 priorities.[13] Data relating to the PSP are available upon reasonable request to IIH UK (www.iih.org.uk).

Results

The prioritisation survey generated 356 responses (figure 1). Demographic data for those with IIH is provided in supplemental table 4 and details of HCP specialisms in supplemental table 5. Of the 2,405 generated uncertainties, 140 were out of scope. The resulting 2,265 were grouped into 64 indicative questions. Sixteen were deemed to be already known or unanswerable by research, leaving 48 questions for presentation in the interim survey. Responses from 512 people were collected in a ratio of 4:1 people with IIH, friends and carers to HCP.

A final list of 26 prioritised questions was generated from the analysis of the interim survey, which included the top 10 for both groups (supplemental table 6). The commonest themes from non-HPC was why the disease develops and progresses; hormonal causes and female predominance; and the conditions associated with IIH. For HCP education; the utility of

For peer review only - http://bmjopen.bmj.com/site/about/guidelines.xhtml

For peer review only

biomarkers; and biological mechanisms of headache were the commonest. At the consensus workshop the top 10 priorities were agreed (figure 2; supplemental table 7).

Discussion

Understanding the most relevant research projects to fund can be challenging. It is imperative that the topics identified in a disease area have the utmost relevance to patients affected by the disease and recognised by clinicians that have clear understanding of the clinical entity. We have undertaken a JLA PSP to establish the top 10 research areas for IIH.

The IIH JLA PSP was funded by IIH UK and set up those who have an active collaboration to improve care for people with IIH.[15] The principles and structured process outlined by the JLA was adhered to steadfastly throughout.[13] All data was maintained in a manner that could be tracked back at any point to the original questions and demographic source; this provided transparency.

Within the feedback people with IIH voiced that they felt their opinions were often not heard. There was a good response rate from all groups when considering how rare IIH is. Submissions with low duplication rates were not removed, a process which can introduce bias. All submitted uncertainties were considered in the long list if they were determined to be known unknowns, including those asked by a single respondent. The data analysis followed standard protocols, though it was complicated by the use of multiple questions in the initial survey (supplemental table 3) as each respondent could appear in up to seven separate initial categories.

For peer review only - http://bmjopen.bmj.com/site/about/guidelines.xhtml

Despite the use of identification codes, the multi-level process meant that the number of individuals contributing to the final data set could not be reasonably calculated. The project took 18 months and surveys were closed on schedule, leaving the possibility that this happened before the maximum number of respondents could contribute.

A major challenge for the IIH PSP steering group was to engage all the relevant HCP (namely neurologists, ophthalmologists, neurosurgeons, radiologists, orthoptists). The speciality diversity brought strength to the process and allowed for a broad inclusion, however during the final selection for the top ten, clinicians were clearly polarised by their individual specialism. There are a number of surgical treatments for fulminant visual loss in the form of CSF diversion, as directed by neurosurgeons, and optic nerve sheath fenestration, as performed by ophthalmic surgeons. [16] More recently interventional radiologists have performed venous sinus stenting for IIH. [17] Physicians (both neurologists and ophthalmologists) use weight loss and medical therapies such as acetazolamide and topiramate. [1,18] This mix of specialism and approach in certain patient groups, i.e. those at threat of visual loss or those with chronic headache, led to expectantly different opinions: for example, surgeons were keen for novel interventions, whereas physicians were promoting better medical therapies.

At the interim survey it was clear there was a discrepancy between the non-HCP and HCP in their most popular themes, with patients keen for research into the aetiology, and HCP

For peer review only - http://bmjopen.bmj.com/site/about/guidelines.xhtml

more commonly ranked education, biomarkers and the pathological mechanisms driving headache. The top priority of the patients' group at the interim survey, was the same as the final result of the consensus workshop.

Some differing opinions between non-HCP and HCP were expressed at the workshop. One issue was surrounding weight loss, seen by physicians as the only disease modifiable therapy and so a high priority for further understanding. This was a highly sensitive issue among the patients and carers present who voiced that it was not considered so important by patients. During the workshop a collective decision was made to have a wide scope within the top 10 areas. If a topic was already featured high within the list, questions that contained a similar theme were purposely voted lower. For example, weight loss, the longer more detailed question was ranked higher than the question regarding bariatric surgery, with the reasoning that it could be answered not only by the weight loss question but also by number 10: the intervention question. For this reason, no further ranking below the top 10 should be published. Of note two areas that did not feature in the top 10, namely multidisciplinary clinics and an education program. They were scored as important during the interim survey, particularly by HPC. The consensus workshop delegates agreed that although these are highly important, the PSP is intended to inform grant bodies who fund research and these areas were universally accepted to require improvement.

The IIH PSP has been an opportunity to understand the areas that are important to all. The primary topic of underlying aetiology requires work both clinically and within the basic laboratory research. Another key area highlighted by this PSP is that of mechanisms of

For peer review only - http://bmjopen.bmj.com/site/about/guidelines.xhtml

headache in IIH. There is increasing evidence regarding the phenotype of the IIH headache, which is challenging tradition regarding the raised ICP headache. [19,20] Future work should explore novel therapies for headache in IIH, which is the key driver in lowering quality of life in this patient cohort. [11] The PSP has the potential to influence the research agenda and consequently in time all area of management, from medical to surgical interventions for this currently idiopathic disease.

For peer review only

For peer review only - http://bmjopen.bmj.com/site/about/guidelines.xhtml

References

1. Mollan SP, Davies B, Silver NC, et al., Idiopathic intracranial hypertension: consensus guidelines on management. J Neurol Neurosurg Psychiatry 2018; 89(10):1088-1100
2. Piper RJ, Kalyvas AV, Young AM et al. Interventions for idiopathic intracranial hypertension. Cochrane Database Syst Rev, 2015(8): p. CD003434.
3. Ball AK, Howman A, Wheatley K et al. A randomised controlled trial of treatment for idiopathic intracranial hypertension. J Neurol, 2011. 258(5): p. 874-81.
4. Committee, N.I.I.H.S.G.W., et al., Effect of acetazolamide on visual function in patients with idiopathic intracranial hypertension and mild visual loss: the idiopathic intracranial hypertension treatment trial. JAMA, 2014. 311(16): p. 1641-51.
5. Markey KA, Ottridge R, Mitchell JL et al. Assessing the Efficacy and Safety of an 11β-Hydroxysteroid Dehydrogenase Type 1 Inhibitor (AZD4017) in the Idiopathic Intracranial Hypertension Drug Trial, IIH:DT: Clinical Methods and Design for a Phase II Randomized Controlled Trial. JMIR Research Protocols, 2017. 6(9): p. e181.
6. Ottridge R, Mollan SP, Botfield H et al. Randomised controlled trial of bariatric surgery versus a community weight loss programme for the sustained treatment of idiopathic intracranial hypertension: the Idiopathic Intracranial Hypertension Weight Trial (IIH:WT) protocol. BMJ Open, 2017. 7(9).
7. Markey KA, Mollan SP, Jensen RH, Sinclair AJ. Understanding idiopathic intracranial hypertension: mechanisms, management, and future directions. Lancet Neurol, 2016. 15(1):78-91.
8. Mollan SP, Ali F, Hassan-Smith G et al. Evolving evidence in adult idiopathic intracranial hypertension: pathophysiology and management. J Neurol Neurosurg Psychiatry 2016; 87(9):982-92.
9. Hornby C, Mollan SP, Botfield H et al. Metabolic Concepts in Idiopathic Intracranial Hypertension and Their Potential for Therapeutic Intervention. J Neuroophthalmol. 2018;38(4):522-530.
10. Mollan SP, Aguiar M, Evison F, Frew E, Sinclair AJ. The expanding burden of idiopathic intracranial hypertension. Eye (Lond). 2018 Oct 24. doi: 10.1038/s41433-018-0238-5.
11. Mulla Y, Markey KA, Woolley RL, et al. Headache determines quality of life in idiopathic intracranial hypertension. The Journal of Headache and Pain. 2015;16:45. doi:10.1186/s10194-015-0521-9.
12. Friesner D, Rosenman R, Lobb BM, Tanne, E. Idiopathic intracranial hypertension in the USA: the role of obesity in establishing prevalence and healthcare costs. Obesity Reviews 2011; 12: e372–e380
13. Cowan, K. and S. Oliver, The James Lind Alliance Guidebook. 2016: http://www.jla.nihr.ac.uk/jla-guidebook/.
14. Sheldon CA, Paley GL, Xiao R, et al. Pediatric Idiopathic Intracranial Hypertension: Age, Gender, and Anthropometric Features at Diagnosis in a Large, Retrospective, Multisite Cohort. Ophthalmology 2016;123(11):2424-2431.
15. Scotton WJ, Mollan SP, Walters T, et al. Characterising the patient experience of diagnostic lumbar puncture in idiopathic intracranial hypertension: a cross-sectional online survey. BMJ Open. 2018;8(5):e020445. doi:10.1136/bmjopen-2017-020445.
16. Spitze A, Malik A, Al-Zubidi N, et al. Optic Nerve Sheath Fenestration vs Cerebrospinal Diversion Procedures: What Is the Preferred Surgical Procedure for

For peer review only - http://bmjopen.bmj.com/site/about/guidelines.xhtml

the Treatment of Idiopathic Intracranial Hypertension Failing Maximum Medical Therapy? Journal of Neuro-Ophthalmology 2013;(33): 183–188.

17. Saber H, Lewis W, Sadeghi M, Rajah G, Narayanan S. Stent Survival and Stent-Adjacent Stenosis Rates following Venous Sinus Stenting for Idiopathic Intracranial Hypertension: A Systematic Review and Meta-Analysis. Interv Neurol. 2018;7(6):490-500.
18. Hoffmann J, Mollan SP, Paemeleire K, et al. European headache federation guideline on idiopathic intracranial hypertension. J Headache Pain. 2018;19(1):93.
19. Mollan SP, Hoffman J, Sinclair AJ. Advances in the understanding of headache in idiopathic intracranial hypertension. Curr Opin Neurol 2018, 31:000–000 DOI:10.1097/WCO.0000000000000651.
20. Mollan SP, Spitzer D, Nicholl DJ. Raised intracranial pressure in those presenting with headache. BMJ 2018 Oct 4;363:k3252.

For peer review only

For peer review only - http://bmjopen.bmj.com/site/about/guidelines.xhtml

Running title: PSP IIH top ten

Acknowledgements:

We would like to acknowledge all the people who contributed to the surveys. We would also like to acknowledge the support from the partner organisations: Association of British Neurologists; British Association for the Study of Headache; British and Irish Orthoptic Society; Fight for Sight – The Eye Research Charity; The Royal College of Ophthalmologists; The Society of British Neurological Surgeons CSF group; Shine; The Neurological Alliance and The United Kingdom Neuro-Ophthalmology Special Interest Group.

Conflicts of Interest

No authors contributing have a conflict of interest in the subject matter.

Funding

AJS is funded by an NIHR Clinician Scientist Fellowship (NIHR-CS-011-028) and by the Medical Research Council, UK (MR/K015184/1). The JLS IIH PSP project was funded by IIH UK.

Data Sharing statement

No additional data is available.

Contributor Statement

Mollan SP: interpretation of the survey results; drafting and review of the manuscript.
Hemmings K: PSP patient lead; administration of both surveys; drafting and review of the manuscript.
Herd C: literature review; independent information specialist; drafting and review of the manuscript.
Denton A**:** critical review of the manuscript.
Williamson M: organisation of the consensus final workshop; and critical review of the manuscript.
Sinclair AJ: PSP clinical lead; interpretation of the survey results and critical review of the manuscript.

All authors were steering group members and have read and approved the final manuscript.

Legend to figures:

Figure 1: Consort diagram and details of the JLA IIH PSP
Figure 2: Final Top 10 ranked uncertainties for the concerning the treatment and management of people with Idiopathic Intracranial Hypertension

For peer review only - http://bmjopen.bmj.com/site/about/guidelines.xhtml

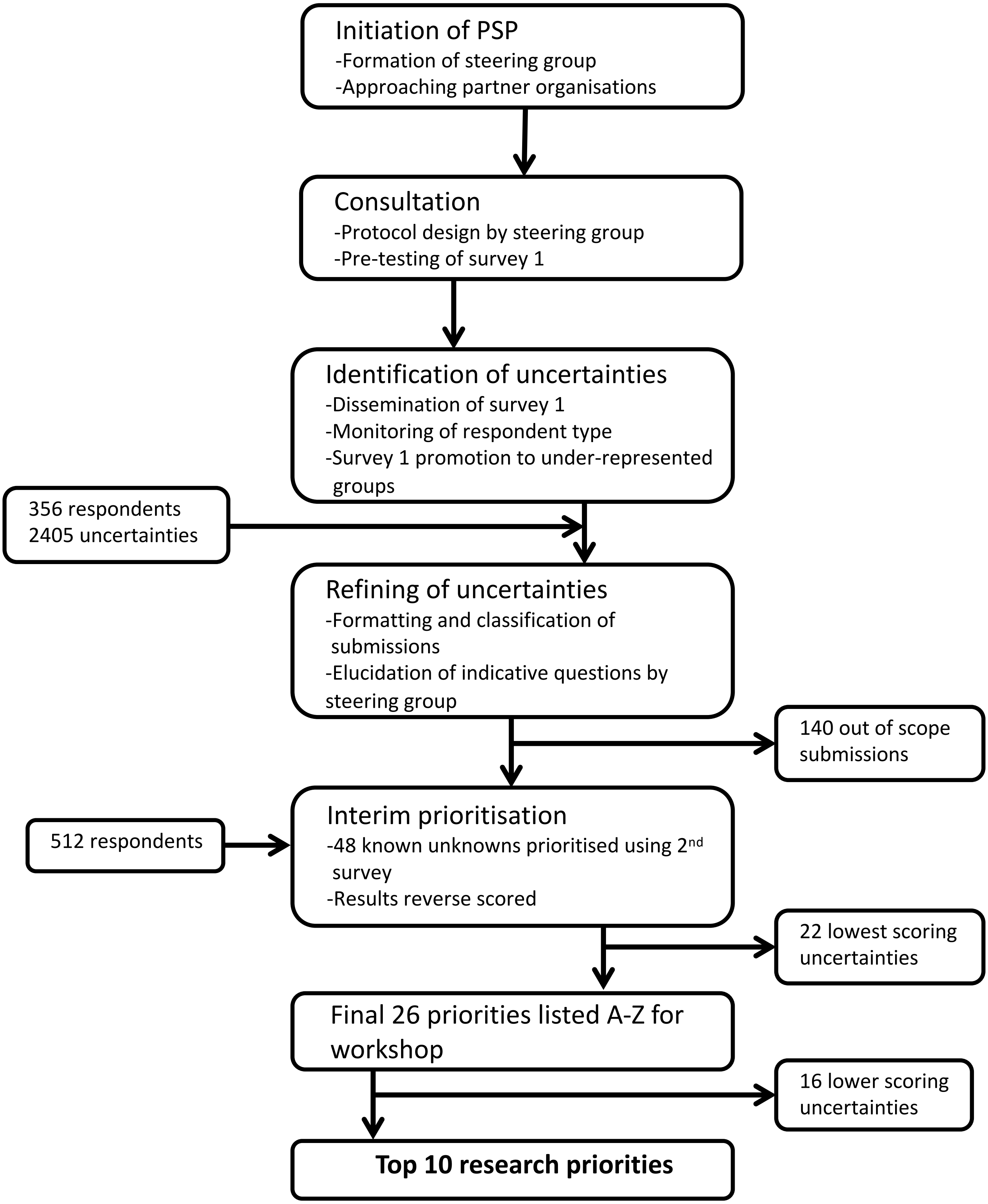
Initiation of PSP
-Formation of steering group
-Approaching partner organisations
Consultation
-Protocol design by steering group
-Pre-testing of survey 1
Identification of uncertainties
-Dissemination of survey 1
-Monitoring of respondent type
-Survey 1 promotion to under-represented groups
356 respondents
2405 uncertainties
Refining of uncertainties
-Formatting and classification of submissions
-Elucidation of indicative questions by steering group
140 out of scope submissions
Interim prioritisation
-48 known unknowns prioritised using 2nd survey
-Results reverse scored
512 respondents
22 lowest scoring uncertainties
Final 26 priorities listed A-Z for workshop
16 lower scoring uncertainties
Top 10 research priorities

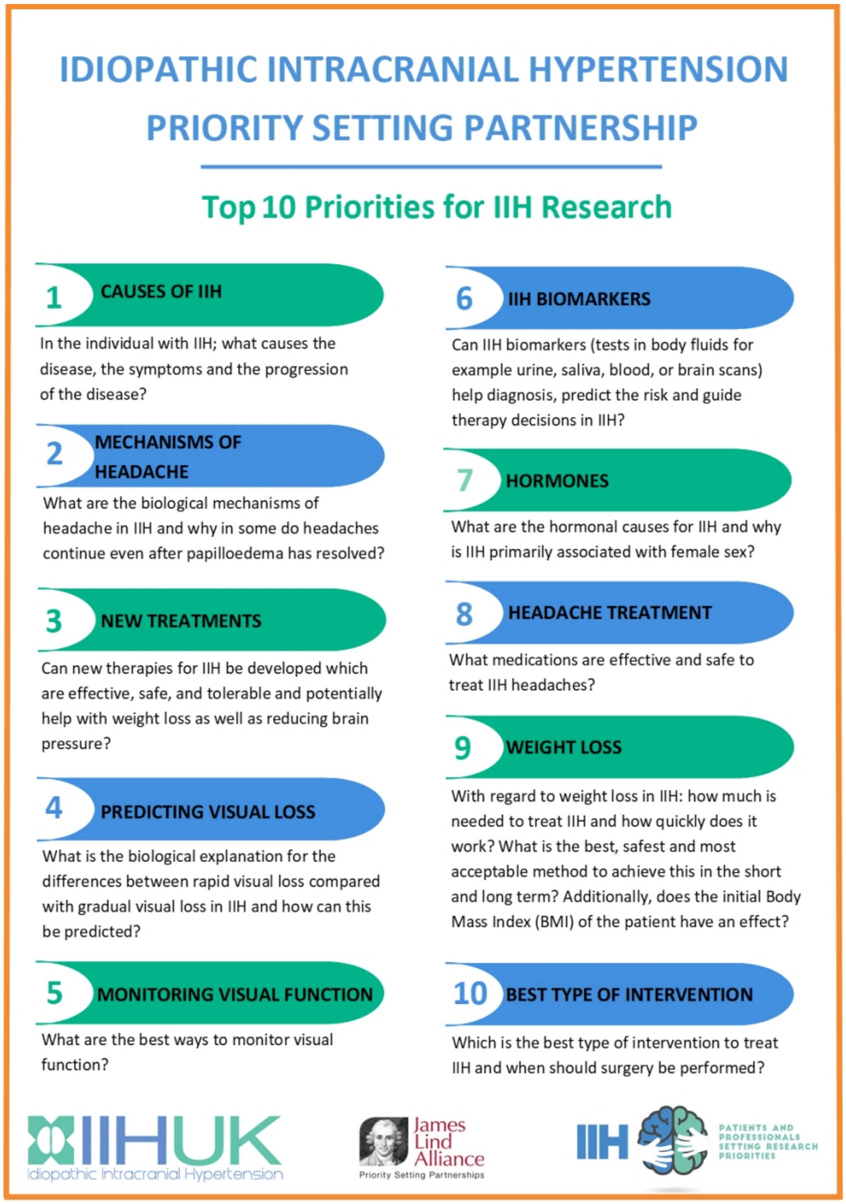

Figure 2: The top ten research priorities in IIH

192x267mm (150 x 150 DPI)

Supplemental table 1: The steering group members and their role

Person	Role
Krystal Hemmings	IIH UK Research representative and PSP patient led
Alex Sinclair	Clinical lead and neurologist
Michelle Williamson	IIH UK Chair trustee, project coordinator and carer
Clare Herd	Information specialist
Martin Plowright	IIH patient
Norma-Ann Dan	IIH UK patient representative
Amanda Denton	IIH UK patient representative
Rachel Bennett	IIH patient
Jayne Best	Neuro-Ophthalmologist
Arun Chandran	Neuro-radiologist
Julie Edwards	Headache nurse specialist
Anita Krishnan	Neurologist
Kamal Mahawar	Bariatric surgeon
Susan Mollan	Neuro-Ophthalmologist
Caroline Rick	Trial methodologist
Ahmed Toma	Neurosurgeon

For peer review only

Supplemental table 2: The prioritisation survey was designed using Qualtrics software (www.qualtrics.com) and responses were requested to the following seven questions:

1.	What questions do you have about how the diagnosis of IIH is made?
2.	What questions do you have about why people get IIH?
3.	What questions do you have about the management of vision in IIH?
4.	What questions do you have about the management of headache in IIH?
5.	What questions do you have about weight management in IIH?
6.	What questions do you have about care provision for patients with IIH? (e.g. General Practice, inpatient, outpatient care)
7.	Do you have any other questions about IIH that you feel are important but do not fall into the categories above?

For peer review only

Supplemental table 3: Partner organisations in alphabetical order

ABN - Association of British Neurologists
BASH - British Association for the Study of Headache
BIOS - British and Irish Orthoptic Society
Fight for Sight – The Eye Research Charity
RCOphth – The Royal College of Ophthalmologists
SBNS CSF subgroup - The Society of British Neurological Surgeons
Shine – Spina bifida, Hydrocephalus, Information, Networking, Equality
The Neurological Alliance
UKNOSIG - The United Kingdom Neuro-Ophthalmology Special Interest Group

For peer review only

Supplementary Table 4: Characteristics of participants with IIH of first survey

Number	180
Female (%)	96
Median age (years)	35
Ethnicity (%)	
White	92
Black or Asian	3
Multiple ethnic backgrounds	4
Not stated	1

For peer review only

Supplementary Table 5: Declared specialism of the healthcare professionals in first survey

Declared specialism of the healthcare professional	% of respondents
Neurologist	45
Ophthalmologist	11
Neurosurgeon	10
Neuro-Ophthalmologist	9
Other	8
Trainee	6
Bariatric Surgeon	3
General Practitioner	3
Nurse	2
Neuroradiologist	1
Orthoptist	1
Not declared	1

For peer review only

Supplemental table 6: 26 Questions for IIH PSP final workshop in alphabetical order

Are multidisciplinary clinics (joint clinics of neurology, ophthalmology, neurosurgery, dietetics and specialist nurses etc.) clinically and cost effective for the management of IIH and would they improve patient experience?
Are non-invasive intracranial pressure (ICP) measurements accurate and clinically useful?
Can IIH biomarkers (tests in body fluids for example urine, saliva, blood, or brain scans) help diagnosis, predict the risk and guide therapy decisions in IIH?
Can novel therapies for IIH be developed which are effective, safe, and tolerable and potentially help with weight loss as well as reducing brain pressure?
Do lumbar punctures (LPs) have long-term safety complications?
Do the benefits of the drug treatments for IIH outweigh the side effects?
How big is the impact of headache in IIH (how severe are headaches, how often do they occur, how many years do they continue for and how do they impact patients quality of life)?
Is bariatric surgery effective in IIH and at what point in the disease should it be performed?
Is cerebral venous stenosis the cause or consequence of IIH?
Is IIH a lifelong condition?
Is IIH caused by increased production or lack of cerebral spinal fluid (CSF) absorption?
Is there a genetic cause of IIH?
Is there a single or are there multiple causes for IIH?
What are the best ways to monitor visual function?
What are the biological mechanisms of headache in IIH and why in some do headaches continue even after papilloedema has resolved?
What are the hormonal causes for IIH and why is IIH primarily associated with female gender?
What are the triggers for periods of high intracranial pressure (ICP) in people with IIH?
What is happening in the body of a person with IIH which causes the development of the disease, the symptoms and the progression of the disease?
What is the biological explanation for the differences between rapid visual loss compared with gradual visual loss in IIH and how can this be predicted?
What medications are effective and safe to treat IIH headaches?
What other conditions / features are associated with IIH (e.g. depression, sleep apnoea, endocrine disorders, cognition, nerve pain)?
Which is the best type of surgery to treat IIH and when should surgery be performed?
Why do people get IIH without papilloedema (IIHWOP) and how should this be treated?
Why is obesity a risk factor for IIH in women and why is this not the case in men?
With regard to weight loss in IIH: how much is needed to treat IIH and how quickly does it work? What is the best, safest and most acceptable method to achieve this in the short and long term? Additionally, does the initial Body Mass Index (BMI) of the patient have an effect?
Would an education program for health care professionals and patients with IIH improve care and disease experience for IIH patients?

For peer review only

For peer review only

Supplemental Table 7: Final Top 10 ranked uncertainties for the concerning the treatment and management of people with Idiopathic Intracranial Hypertension

Ranking	Research priority
1	In the individual with IIH; what causes the disease, the symptoms and the progression of the disease?
2	What are the biological mechanisms of headache in IIH and why in some do headaches continue even after papilloedema has resolved?
3	Can new medical therapies for IIH be developed which are effective, safe, and tolerable and potentially help with weight loss as well as reducing brain pressure?
4	What is the biological explanation for the differences between rapid visual loss compared with gradual visual loss in IIH and how can this be predicted?
5	What are the best ways to monitor visual function?
6	Can IIH biomarkers (tests in body fluids for example urine, saliva, blood, or brain scans) help diagnosis, predict the risk and guide therapy decisions in IIH?
7	What are the hormonal causes for IIH and why is IIH primarily associated with female sex?
8	What medications are effective and safe to treat IIH headaches?
9	With regard to weight loss in IIH: how much is needed to treat IIH and how quickly does it work? What is the best, safest and most acceptable method to achieve this in the short and long term? Additionally, does the initial Body Mass Index (BMI) of the patient have an effect?
10	Which is the best type of intervention to treat IIH and when should surgery be performed?

For peer review only

BMJ Open

What are the research priorities for idiopathic intracranial hypertension? A priority setting partnership between patients and health care professionals.

Journal:	*BMJ Open*
Manuscript ID	bmjopen-2018-026573.R2
Article Type:	Research
Date Submitted by the Author:	23-Jan-2019
Complete List of Authors:	Mollan, Susan; University Hospitals Birmingham NHS Foundation Trust<br>Hemmings, Krystal; IIH UK<br>Herd, Clare; University of Birmingham, Institute of Applied Health Research<br>Denton, Amanda; IIH UK<br>Williamson, Shelley; Idiopathic Intracranial Hypertension UK,<br>Sinclair, AJ; University of Birmingham, Metabolic Neurology; University Hospitals Birmingham
**Primary Subject Heading**:	Neurology
Secondary Subject Heading:	Ophthalmology, Research methods, Patient-centred medicine
Keywords:	Idiopathic Intracranial Hypertension, Priority setting, Research priorities, Patient involvement, Service-user involvement, NEUROLOGY

SCHOLARONE™
Manuscripts

Running title: PSP IIH top ten

What are the research priorities for idiopathic intracranial hypertension? A priority setting partnership between patients and health care professionals.

Mollan SP* (http://orcid.org/0000-0002-6314-4437) [1]
Hemmings K* [2]
Herd C [3]
Denton A [2]
Williamson S [2]
Sinclair AJ (orcid.org/0000-0003-2777-5132)[4,5,6]

1. Birmingham Neuro-Ophthalmology, Queen Elizabeth Hospital, Birmingham, UK
2. IIH UK, Washington, Tyne and Wear, UK
3. Institute of Applied Health Research, University of Birmingham, Edgbaston, UK
4. Metabolic Neurology, Institute of Metabolism and Systems Research, University of Birmingham, Birmingham, United Kingdom (UK)
5. Department of Neurology, University Hospitals Birmingham, Queen Elizabeth Hospital, Birmingham, UK
6. Centre for Endocrinology, Diabetes and Metabolism, Birmingham Health Partners, Birmingham, UK

* Both authors should be considered joint first author, as they contributed equally to the paper.

Abstract word count: 200
Word count: 1873
References: 19

Corresponding Author: Dr Alexandra J Sinclair, Metabolic Neurology, Institute of Metabolism and Systems Research, University of Birmingham, Birmingham B15 2TT, UK; a.b.sinclair@bham.ac.uk

Key Words: Idiopathic Intracranial Hypertension; Priority setting; Research priorities; Patient involvement; Patient and carer involvement; PPI; Service-user involvement; Impact; Research agenda

For peer review only - http://bmjopen.bmj.com/site/about/guidelines.xhtml

Running title: PSP IIH top ten

Abstract

Objective

Idiopathic Intracranial Hypertension (IIH) is under-researched and the aim was to determine the top 10 research priorities for this disease.

Design

A modified nominal group technique was used to engage participants who had experience of IIH.

Setting

This James Lind Alliance Priority Setting Partnership was commissioned by IIH UK, a charity.

Participants

People with IIH, carers, family and friends, and healthcare professionals participated in two rounds of surveys to identify unique research questions unanswered by current evidence. The most popular 26 uncertainties were presented to stakeholders who then agreed the top 10 topics.

Results

The top 10 research priorities for IIH included aetiology of IIH; the pathological mechanisms of headache in IIH; new treatments in IIH; the difference between acute and gradual visual loss; the best ways to monitor visual function; biomarkers of the disease; hormonal causes of IIH; drug therapies for treatment of headache; weight loss and its role in IIH; and finally, the best intervention to treat IIH and when should surgery be performed.

Conclusions

This priority setting encouraged people with direct experience of IIH to collectively identify critical gaps in the existing evidence. The overarching research aspiration was to understand the aetiology and management of IIH.

For peer review only

Running title: PSP IIH top ten

Strengths and limitations

- This is the first collaboration of patients, carers and clinicians with experience of Idiopathic Intracranial Hypertension (IIH) to achieve consensus on the priorities for future research.
- The James Lind Alliance (JLA) methods are patient centred and give funding bodies an unbiased agenda for research in IIH.
- Using online surveys as the main method for gathering questions for this Priority Setting Partnership (PSP) may mean that not all those with experience of IIH were aware or able to participate in the process.
- It is conceivable that possibly all the research questions gathered are not exhaustive.
- While the JLA process and IIH PSP study recommend those research priorities that are important, there is no guarantee of research funding.

For peer review only

Running title: PSP IIH top ten

Introduction

Clinical uncertainty in Idiopathic Intracranial Hypertension (IIH) is evident, with the first consensus guidelines for investigation and management stating uncertainties in every aspect of the disease .[1] The 2015 Cochrane review concluded that there is a lack of evidence to guide pharmacological treatment.[2] There are few published randomised clinical trials (RCTs)[3,4] and a small number of ongoing trials.[5,6] Research is infrequent due to the rarity of the IIH [7,8] and the lack of understanding of the underlying pathology.[9]

IIH predominantly affects overweight women of childbearing age with the incidence of the disease documented to be rising [10] with the increasing prevalence of obesity.[7,8] In those with severely affected vision, surgery may be indicated.[1] For the majority, it can be a chronic condition, with headaches impacting on the quality of life of patients, [11] and an economic burden [10,12].

Understanding where research should be directed was a priority for IIH UK, the leading charity for IIH in the United Kingdom (UK). The James Lind Alliance (JLA), a UK National Institute for Health Research-supported initiative, aims to provide a transparent process that enables patients and healthcare professionals (HCP) to work together to agree on the most important uncertainties to inform the research agenda. The aim of this IIH priority setting partnership (PSP), was to identify gaps in knowledge that matter most to key stakeholders (patients, carers and clinicians), and to indicate where future funding should be placed.

For peer review only - http://bmjopen.bmj.com/site/about/guidelines.xhtml

Running title: PSP IIH top ten

Methods

IIH PSP process

The University of Birmingham, UK, acted as academic partner to the IIH PSP and the process was led by the IIH UK research representative, in collaboration with the James Lind Alliance (JLA, www.jla.nihr.ac.uk). A steering group with representation from IIH UK, patients and all the major specialities associated with IIH plus an independent information specialist oversaw the process (supplemental table 1). In February 2017, key organisations accepted the invitation to become partners. They included the Association of British Neurologists; the British Association for the Study of Headache; the British and Irish Orthoptic Society; Fight for Sight; The Royal College of Ophthalmologists; The Society of British Neurological Surgeons CSF group; Shine; Neurological Alliance and the United Kingdom Neuro-Ophthalmology Special Interest Group (supplemental table 2). The PSP stages were broadly based on the four step process developed by the JLA (figure 1).[13]

This PSP was concerned with adult IIH only and any responses exclusively relating to children were excluded. There was limited funding for the project, and including the paediatric population would have required funding for two different work streams. It is well documented the expectantly different phenotype between adult and those prepubescent children with IIH.[14] However responses were not limited by those who submitted, and hence those with children with IIH are likely to be included. Indeed, at the final stakeholder meeting there was representation from carers of children with IIH. Responses concerning the classification of the disease, healthcare funding/entitlements, or statements without a discernible question were excluded.

For peer review only - http://bmjopen.bmj.com/site/about/guidelines.xhtml

Running title: PSP IIH top ten

The prioritisation survey questions were constructed (supplemental table 2) by the steering group, aided by the first guidelines in IIH where uncertainties exist around the diagnosis, investigation and management.[1] This first survey was advertised by partners (supplemental table 3), IIH UK and steering group members. All responses were refined to understandable 'uncertainties' with the exception of those considered to be 'out of scope'. These were categorised using the UK Clinical Research Collaboration Health Research Classification System, sorted into themes and then formulated into indicative questions by steering group members, working in groups with at least one HCP and one patient representative. A literature search was conducted with the electronic databases CENTRAL, Embase and Medline searched from inception to March 2018 for systematic reviews using strategies based on those used by Piper *at al*. [2]. The "known knowns" with reference to the appropriate literature and duplicate questions were removed. Questions were amalgamated when practical to do so. The long list was then verified by the PSP lead and discussions were held with the wider steering group if disagreements occurred.

The known unknowns were then used for the interim survey. Respondents ranked the questions, returning their top ten. The rankings were reverse scored and the total scores for the two groups: individuals with IIH, friends, or carers; and HCP, were calculated separately to ensure an equal weighting. The most popular 26 questions were then taken forward, which included the top 10 for both groups, to the final workshop, with the aim of consensus on the top 10 priorities.[13] Data relating to the PSP are available upon reasonable request to IIH UK (www.iih.org.uk).

For peer review only - http://bmjopen.bmj.com/site/about/guidelines.xhtml

Ethics

The IIH UK internal review board formally reviewed the project and further ethical approval was not required. All data was anonymised and sent to the information specialist at the University of Birmingham for processing. Each participant gave their consent for the anonymised data to be used when entering their opinions through the online surveys.

Patient and public involvement

This research priority partnership was established by IIH UK, a charity that is run by carers and people with IIH. At each stage of the JLA process, patients and carers were equal collaborators in the design and decisions including the survey design and piloting, survey participation and the final workshop. They disseminated the surveys on the charity website and via social media. All participants were able to indicate a desire for further involvement and for information about the results.

Results

The prioritisation survey generated 356 responses (figure 1). Demographic data for those with IIH is provided in supplemental table 4 and details of HCP specialisms in supplemental table 5. Of the 2,405 generated uncertainties, 140 were out of scope. The resulting 2,265 were grouped into 64 indicative questions. Sixteen were deemed to be already known or unanswerable by research, leaving 48 questions for presentation in the interim survey. Responses from 512 people were collected in a ratio of 4:1 people with IIH, friends and carers to HCP.

For peer review only - http://bmjopen.bmj.com/site/about/guidelines.xhtml

A final list of 26 prioritised questions was generated from the analysis of the interim survey, which included the top 10 for both groups (supplemental table 6). The commonest themes from non-HPC was why the disease develops and progresses; hormonal causes and female predominance; and the conditions associated with IIH. For HCP education; the utility of biomarkers; and biological mechanisms of headache were the commonest. At the consensus workshop the top 10 priorities were agreed (figure 2; supplemental table 7).

For peer review only

Discussion

Understanding the most relevant research projects to fund can be challenging. It is imperative that the topics identified in a disease area have the utmost relevance to patients affected by the disease and recognised by clinicians that have clear understanding of the clinical entity. We have undertaken a JLA PSP to establish the top 10 research areas for IIH.

The IIH JLA PSP was funded by IIH UK and set up those who have an active collaboration to improve care for people with IIH.[15] The principles and structured process outlined by the JLA was adhered to steadfastly throughout.[13] All data was maintained in a manner that could be tracked back at any point to the original questions and demographic source; this provided transparency.

A major challenge for the IIH PSP steering group was to engage all the relevant HCP (namely neurologists, ophthalmologists, neurosurgeons, radiologists, orthoptists). The speciality

For peer review only - http://bmjopen.bmj.com/site/about/guidelines.xhtml

diversity brought strength to the process and allowed for a broad inclusion, however during the final selection for the top ten, clinicians were clearly polarised by their individual specialism. There are a number of surgical treatments for fulminant visual loss in the form of CSF diversion, as directed by neurosurgeons, and optic nerve sheath fenestration, as performed by ophthalmic surgeons. [16] More recently interventional radiologists have performed venous sinus stenting for IIH. [17] Physicians (both neurologists and ophthalmologists) use weight loss and medical therapies such as acetazolamide and topiramate. [1,18] This mix of specialism and approach in certain patient groups, i.e. those at threat of visual loss or those with chronic headache, led to expectantly different opinions: for example, surgeons were keen for novel interventions, whereas physicians were promoting better medical therapies.

At the interim survey it was clear there was a discrepancy between the non-HCP and HCP in their most popular themes, with patients keen for research into the aetiology, and HCP more commonly ranked education, biomarkers and the pathological mechanisms driving headache. The top priority of the patients' group at the interim survey, was the same as the final result of the consensus workshop.

Some differing opinions between non-HCP and HCP were expressed at the workshop. One issue was surrounding weight loss, seen by physicians as the only disease modifiable therapy and so a high priority for further understanding. This was a highly sensitive issue among the patients and carers present who voiced that it was not considered so important by patients. During the workshop a collective decision was made to have a wide scope

For peer review only

within the top 10 areas. If a topic was already featured high within the list, questions that contained a similar theme were purposely voted lower. For example, weight loss, the longer more detailed question was ranked higher than the question regarding bariatric surgery, with the reasoning that it could be answered not only by the weight loss question but also by number 10: the intervention question. For this reason, no further ranking below the top 10 should be published. Of note two areas that did not feature in the top 10, namely multidisciplinary clinics and an education program. They were scored as important during the interim survey, particularly by HPC. The consensus workshop delegates agreed that although these are highly important, the PSP is intended to inform grant bodies who fund research and these areas were universally accepted to require improvement.

Strengths

Within the feedback people with IIH voiced that they felt their opinions were often not heard, therefore the IIH PSP has allowed them a voice. There was a good response rate from all groups when considering how rare IIH is. Submissions with low duplication rates were not removed, a process which can introduce bias. All submitted uncertainties were considered in the long list if they were determined to be known unknowns, including those asked by a single respondent. The data analysis followed standard protocols, though it was complicated by the use of multiple questions in the initial survey (supplemental table 3) as each respondent could appear in up to seven separate initial categories.

Limitations

For peer review only - http://bmjopen.bmj.com/site/about/guidelines.xhtml

Despite the use of identification codes, the multi-level process meant that the number of individuals contributing to the final data set could not be reasonably calculated. The project took 18 months and surveys were closed on schedule, leaving the possibility that this happened before the maximum number of respondents could contribute. Using online surveys as the main method for gathering questions for this Priority Setting Partnership (PSP) may mean that not all those with experience of IIH were aware or able to participate in the process. It is conceivable that possibly all the research questions gathered are not exhaustive. While the JLA process and IIH PSP study recommend those research priorities that are important, there is no guarantee of research funding.

Conclusions

The IIH PSP has been an opportunity to understand the areas that are important to all. The primary topic of underlying aetiology requires work both clinically and within the basic laboratory research. Another key area highlighted by this PSP is that of mechanisms of headache in IIH. There is increasing evidence regarding the phenotype of the IIH headache, which is challenging tradition regarding the raised ICP headache. [19,20] Future work should explore novel therapies for headache in IIH, which is the key driver in lowering quality of life in this patient cohort. [11] The PSP has the potential to influence the research agenda and consequently in time all area of management, from medical to surgical interventions for this currently idiopathic disease.

For peer review only - http://bmjopen.bmj.com/site/about/guidelines.xhtml

References

1. Mollan SP, Davies B, Silver NC, et al., Idiopathic intracranial hypertension: consensus guidelines on management. J Neurol Neurosurg Psychiatry 2018; 89(10):1088-1100
2. Piper RJ, Kalyvas AV, Young AM et al. Interventions for idiopathic intracranial hypertension. Cochrane Database Syst Rev, 2015(8): p. CD003434.
3. Ball AK, Howman A, Wheatley K et al. A randomised controlled trial of treatment for idiopathic intracranial hypertension. J Neurol, 2011. 258(5): p. 874-81.
4. Committee, N.I.I.H.S.G.W., et al., Effect of acetazolamide on visual function in patients with idiopathic intracranial hypertension and mild visual loss: the idiopathic intracranial hypertension treatment trial. JAMA, 2014. 311(16): p. 1641-51.
5. Markey KA, Ottridge R, Mitchell JL et al. Assessing the Efficacy and Safety of an 11β-Hydroxysteroid Dehydrogenase Type 1 Inhibitor (AZD4017) in the Idiopathic Intracranial Hypertension Drug Trial, IIH:DT: Clinical Methods and Design for a Phase II Randomized Controlled Trial. JMIR Research Protocols, 2017. 6(9): p. e181.
6. Ottridge R, Mollan SP, Botfield H et al. Randomised controlled trial of bariatric surgery versus a community weight loss programme for the sustained treatment of idiopathic intracranial hypertension: the Idiopathic Intracranial Hypertension Weight Trial (IIH:WT) protocol. BMJ Open, 2017. 7(9).
7. Markey KA, Mollan SP, Jensen RH, Sinclair AJ. Understanding idiopathic intracranial hypertension: mechanisms, management, and future directions. Lancet Neurol, 2016. 15(1):78-91.
8. Mollan SP, Ali F, Hassan-Smith G et al. Evolving evidence in adult idiopathic intracranial hypertension: pathophysiology and management. J Neurol Neurosurg Psychiatry 2016; 87(9):982-92.
9. Hornby C, Mollan SP, Botfield H et al. Metabolic Concepts in Idiopathic Intracranial Hypertension and Their Potential for Therapeutic Intervention. J Neuroophthalmol. 2018;38(4):522-530.
10. Mollan SP, Aguiar M, Evison F, Frew E, Sinclair AJ. The expanding burden of idiopathic intracranial hypertension. Eye (Lond). 2018 Oct 24. doi: 10.1038/s41433-018-0238-5.
11. Mulla Y, Markey KA, Woolley RL, et al. Headache determines quality of life in idiopathic intracranial hypertension. The Journal of Headache and Pain. 2015;16:45. doi:10.1186/s10194-015-0521-9.
12. Friesner D, Rosenman R, Lobb BM, Tanne, E. Idiopathic intracranial hypertension in the USA: the role of obesity in establishing prevalence and healthcare costs. Obesity Reviews 2011; 12: e372–e380
13. Cowan, K. and S. Oliver, The James Lind Alliance Guidebook. 2016: http://www.jla.nihr.ac.uk/jla-guidebook/.
14. Sheldon CA, Paley GL, Xiao R, et al. Pediatric Idiopathic Intracranial Hypertension: Age, Gender, and Anthropometric Features at Diagnosis in a Large, Retrospective, Multisite Cohort. Ophthalmology 2016;123(11):2424-2431.
15. Scotton WJ, Mollan SP, Walters T, et al. Characterising the patient experience of diagnostic lumbar puncture in idiopathic intracranial hypertension: a cross-sectional online survey. BMJ Open. 2018;8(5):e020445. doi:10.1136/bmjopen-2017-020445.
16. Spitze A, Malik A, Al-Zubidi N, et al. Optic Nerve Sheath Fenestration vs Cerebrospinal Diversion Procedures: What Is the Preferred Surgical Procedure for

For peer review only - http://bmjopen.bmj.com/site/about/guidelines.xhtml

the Treatment of Idiopathic Intracranial Hypertension Failing Maximum Medical Therapy? Journal of Neuro-Ophthalmology 2013;(33): 183–188.

17. Saber H, Lewis W, Sadeghi M, Rajah G, Narayanan S. Stent Survival and Stent-Adjacent Stenosis Rates following Venous Sinus Stenting for Idiopathic Intracranial Hypertension: A Systematic Review and Meta-Analysis. Interv Neurol. 2018;7(6):490-500.
18. Hoffmann J, Mollan SP, Paemeleire K, et al. European headache federation guideline on idiopathic intracranial hypertension. J Headache Pain. 2018;19(1):93.
19. Mollan SP, Hoffman J, Sinclair AJ. Advances in the understanding of headache in idiopathic intracranial hypertension. Curr Opin Neurol 2019; 32(1):92-98 DOI:10.1097/WCO.0000000000000651.
20. Mollan SP, Spitzer D, Nicholl DJ. Raised intracranial pressure in those presenting with headache. BMJ 2018 Oct 4;363:k3252.

For peer review only

For peer review only - http://bmjopen.bmj.com/site/about/guidelines.xhtml

Running title: PSP IIH top ten

Acknowledgements:

We would like to acknowledge all the people who contributed to the surveys. We would also like to acknowledge the support from the partner organisations: Association of British Neurologists; British Association for the Study of Headache; British and Irish Orthoptic Society; Fight for Sight – The Eye Research Charity; The Royal College of Ophthalmologists; The Society of British Neurological Surgeons CSF group; Shine; The Neurological Alliance and The United Kingdom Neuro-Ophthalmology Special Interest Group.

Conflicts of Interest

No authors contributing have a conflict of interest in the subject matter.

Funding

AJS is funded by an NIHR Clinician Scientist Fellowship (NIHR-CS-011-028) and by the Medical Research Council, UK (MR/K015184/1). The JLS IIH PSP project was funded by IIH UK.

Data Sharing statement

No additional data is available.

Contributor Statement

Mollan SP: interpretation of the survey results; drafting and review of the manuscript.
Hemmings K: PSP patient lead; administration of both surveys; drafting and review of the manuscript.
Herd C: literature review; independent information specialist; drafting and review of the manuscript.
Denton A**:** critical review of the manuscript.
Williamson M: organisation of the consensus final workshop; and critical review of the manuscript.
Sinclair AJ: PSP clinical lead; interpretation of the survey results and critical review of the manuscript.

All authors were steering group members and have read and approved the final manuscript.

Legend to figures:

Figure 1: Consort diagram and details of the JLA IIH PSP
Figure 2: Final Top 10 ranked uncertainties for the concerning the treatment and management of people with Idiopathic Intracranial Hypertension

For peer review only - http://bmjopen.bmj.com/site/about/guidelines.xhtml

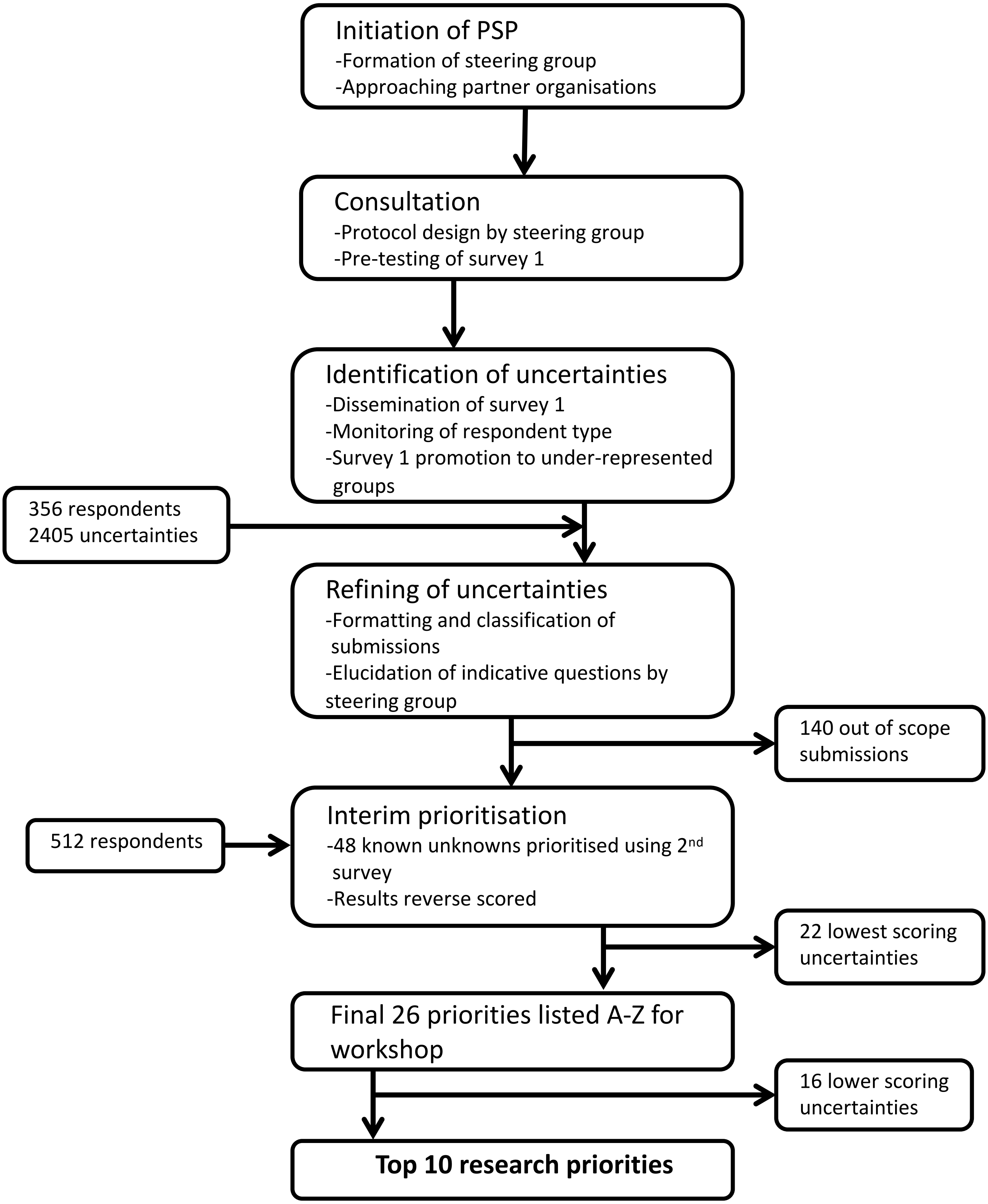
Initiation of PSP
-Formation of steering group
-Approaching partner organisations
Consultation
-Protocol design by steering group
-Pre-testing of survey 1
Identification of uncertainties
-Dissemination of survey 1
-Monitoring of respondent type
-Survey 1 promotion to under-represented groups
356 respondents
2405 uncertainties
Refining of uncertainties
-Formatting and classification of submissions
-Elucidation of indicative questions by steering group
140 out of scope submissions
512 respondents
Interim prioritisation
-48 known unknowns prioritised using 2nd survey
-Results reverse scored
22 lowest scoring uncertainties
Final 26 priorities listed A-Z for workshop
16 lower scoring uncertainties
Top 10 research priorities

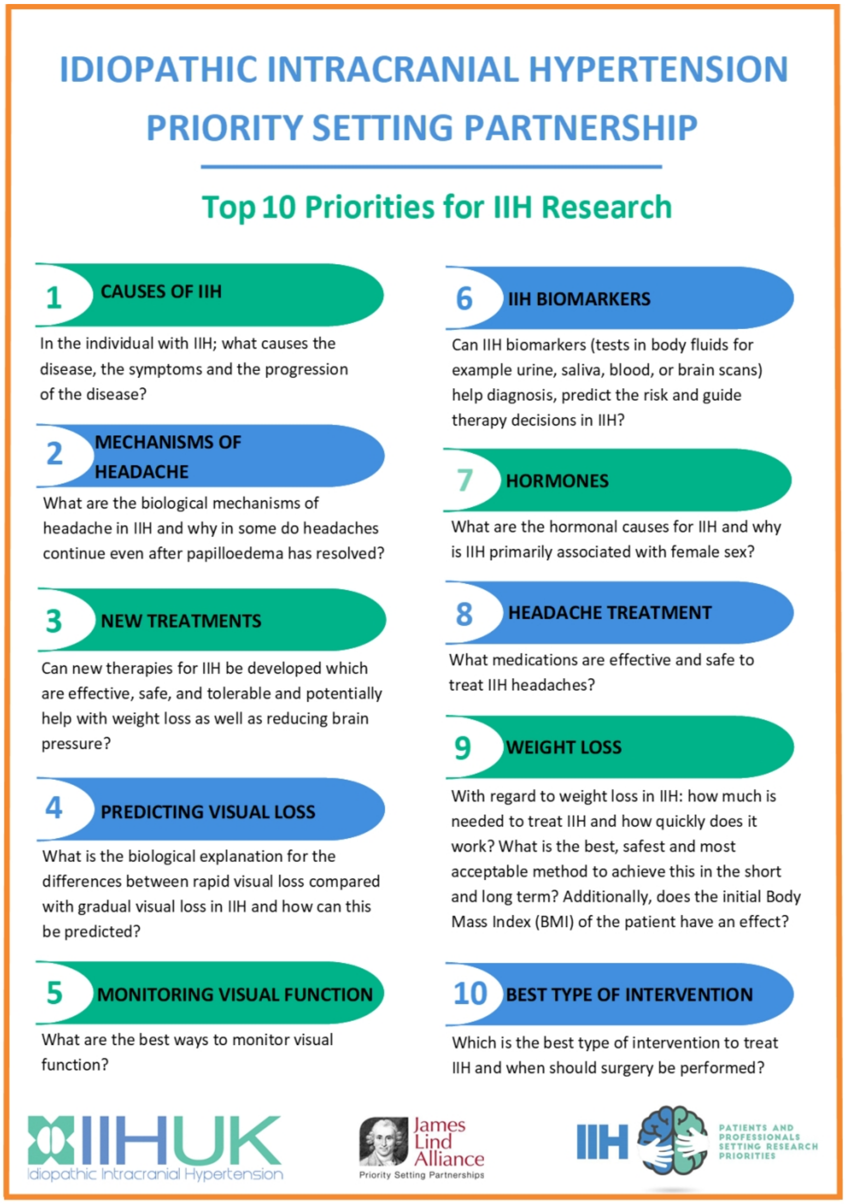

Figure 2: The top ten research priorities in IIH

192x267mm (150 x 150 DPI)

Supplemental table 1: The steering group members and their role

Person	Role
Krystal Hemmings	IIH UK Research representative and PSP patient led
Alex Sinclair	Clinical lead and neurologist
Michelle Williamson	IIH UK Chair trustee, project coordinator and carer
Clare Herd	Information specialist
Martin Plowright	IIH patient
Norma-Ann Dan	IIH UK patient representative
Amanda Denton	IIH UK patient representative
Rachel Bennett	IIH patient
Jayne Best	Neuro-Ophthalmologist
Arun Chandran	Neuro-radiologist
Julie Edwards	Headache nurse specialist
Anita Krishnan	Neurologist
Kamal Mahawar	Bariatric surgeon
Susan Mollan	Neuro-Ophthalmologist
Caroline Rick	Trial methodologist
Ahmed Toma	Neurosurgeon

For peer review only

Supplemental table 2: The prioritisation survey was designed using Qualtrics software (www.qualtrics.com) and responses were requested to the following seven questions:

1. What questions do you have about how the diagnosis of IIH is made?
2. What questions do you have about why people get IIH?
3. What questions do you have about the management of vision in IIH?
4. What questions do you have about the management of headache in IIH?
5. What questions do you have about weight management in IIH?
6. What questions do you have about care provision for patients with IIH? (e.g. General Practice, inpatient, outpatient care)
7. Do you have any other questions about IIH that you feel are important but do not fall into the categories above?

For peer review only

Supplemental table 3: Partner organisations in alphabetical order

ABN - Association of British Neurologists
BASH - British Association for the Study of Headache
BIOS - British and Irish Orthoptic Society
Fight for Sight – The Eye Research Charity
RCOphth – The Royal College of Ophthalmologists
SBNS CSF subgroup - The Society of British Neurological Surgeons
Shine – Spina bifida, Hydrocephalus, Information, Networking, Equality
The Neurological Alliance
UKNOSIG - The United Kingdom Neuro-Ophthalmology Special Interest Group

For peer review only

Supplementary Table 4: Characteristics of participants with IIH of first survey

Number	180
Female (%)	96
Median age (years)	35
Ethnicity (%)	
White	92
Black or Asian	3
Multiple ethnic backgrounds	4
Not stated	1

For peer review only

Supplementary Table 5: Declared specialism of the healthcare professionals in first survey

Declared specialism of the healthcare professional	% of respondents
Neurologist	45
Ophthalmologist	11
Neurosurgeon	10
Neuro-Ophthalmologist	9
Other	8
Trainee	6
Bariatric Surgeon	3
General Practitioner	3
Nurse	2
Neuroradiologist	1
Orthoptist	1
Not declared	1

For peer review only

Supplemental table 6: 26 Questions for IIH PSP final workshop in alphabetical order

Are multidisciplinary clinics (joint clinics of neurology, ophthalmology, neurosurgery, dietetics and specialist nurses etc.) clinically and cost effective for the management of IIH and would they improve patient experience?
Are non-invasive intracranial pressure (ICP) measurements accurate and clinically useful?
Can IIH biomarkers (tests in body fluids for example urine, saliva, blood, or brain scans) help diagnosis, predict the risk and guide therapy decisions in IIH?
Can novel therapies for IIH be developed which are effective, safe, and tolerable and potentially help with weight loss as well as reducing brain pressure?
Do lumbar punctures (LPs) have long-term safety complications?
Do the benefits of the drug treatments for IIH outweigh the side effects?
How big is the impact of headache in IIH (how severe are headaches, how often do they occur, how many years do they continue for and how do they impact patients quality of life)?
Is bariatric surgery effective in IIH and at what point in the disease should it be performed?
Is cerebral venous stenosis the cause or consequence of IIH?
Is IIH a lifelong condition?
Is IIH caused by increased production or lack of cerebral spinal fluid (CSF) absorption?
Is there a genetic cause of IIH?
Is there a single or are there multiple causes for IIH?
What are the best ways to monitor visual function?
What are the biological mechanisms of headache in IIH and why in some do headaches continue even after papilloedema has resolved?
What are the hormonal causes for IIH and why is IIH primarily associated with female gender?
What are the triggers for periods of high intracranial pressure (ICP) in people with IIH?
What is happening in the body of a person with IIH which causes the development of the disease, the symptoms and the progression of the disease?
What is the biological explanation for the differences between rapid visual loss compared with gradual visual loss in IIH and how can this be predicted?
What medications are effective and safe to treat IIH headaches?
What other conditions / features are associated with IIH (e.g. depression, sleep apnoea, endocrine disorders, cognition, nerve pain)?
Which is the best type of surgery to treat IIH and when should surgery be performed?
Why do people get IIH without papilloedema (IIHWOP) and how should this be treated?
Why is obesity a risk factor for IIH in women and why is this not the case in men?
With regard to weight loss in IIH: how much is needed to treat IIH and how quickly does it work? What is the best, safest and most acceptable method to achieve this in the short and long term? Additionally, does the initial Body Mass Index (BMI) of the patient have an effect?
Would an education program for health care professionals and patients with IIH improve care and disease experience for IIH patients?

For peer review only

For peer review only

Supplemental Table 7: Final Top 10 ranked uncertainties for the concerning the treatment and management of people with Idiopathic Intracranial Hypertension

Ranking	Research priority
1	In the individual with IIH; what causes the disease, the symptoms and the progression of the disease?
2	What are the biological mechanisms of headache in IIH and why in some do headaches continue even after papilloedema has resolved?
3	Can new medical therapies for IIH be developed which are effective, safe, and tolerable and potentially help with weight loss as well as reducing brain pressure?
4	What is the biological explanation for the differences between rapid visual loss compared with gradual visual loss in IIH and how can this be predicted?
5	What are the best ways to monitor visual function?
6	Can IIH biomarkers (tests in body fluids for example urine, saliva, blood, or brain scans) help diagnosis, predict the risk and guide therapy decisions in IIH?
7	What are the hormonal causes for IIH and why is IIH primarily associated with female sex?
8	What medications are effective and safe to treat IIH headaches?
9	With regard to weight loss in IIH: how much is needed to treat IIH and how quickly does it work? What is the best, safest and most acceptable method to achieve this in the short and long term? Additionally, does the initial Body Mass Index (BMI) of the patient have an effect?
10	Which is the best type of intervention to treat IIH and when should surgery be performed?

For peer review only

Standards for Reporting Qualitative Research (SRQR)*

http://www.equator-network.org/reporting-guidelines/srqr/

Title and abstract

	Page/line no(s).
**Title** - Concise description of the nature and topic of the study Identifying the study as qualitative or indicating the approach (e.g., ethnography, grounded theory) or data collection methods (e.g., interview, focus group) is recommended	1
**Abstract** - Summary of key elements of the study using the abstract format of the intended publication; typically includes background, purpose, methods, results, and conclusions	2

Introduction

**Problem formulation** - Description and significance of the problem/phenomenon studied; review of relevant theory and empirical work; problem statement	4
**Purpose or research question** - Purpose of the study and specific objectives or questions	4/last paragraph

Methods

**Qualitative approach and research paradigm** - Qualitative approach (e.g., ethnography, grounded theory, case study, phenomenology, narrative research) and guiding theory if appropriate; identifying the research paradigm (e.g., postpositivist, constructivist/ interpretivist) is also recommended; rationale**	5 and 6
**Researcher characteristics and reflexivity** - Researchers' characteristics that may influence the research, including personal attributes, qualifications/experience, relationship with participants, assumptions, and/or presuppositions; potential or actual interaction between researchers' characteristics and the research questions, approach, methods, results, and/or transferability	5 and 7
**Context** - Setting/site and salient contextual factors; rationale**	5
**Sampling strategy** - How and why research participants, documents, or events were selected; criteria for deciding when no further sampling was necessary (e.g., sampling saturation); rationale**	5,6,7
**Ethical issues pertaining to human subjects** - Documentation of approval by an appropriate ethics review board and participant consent, or explanation for lack thereof; other confidentiality and data security issues	7 top paragraph
**Data collection methods** - Types of data collected; details of data collection procedures including (as appropriate) start and stop dates of data collection and analysis, iterative process, triangulation of sources/methods, and modification of procedures in response to evolving study findings; rationale**	5,6,7

For peer review only

For peer review only - http://bmjopen.bmj.com/site/about/guidelines.xhtml

**Data collection instruments and technologies** - Description of instruments (e.g., interview guides, questionnaires) and devices (e.g., audio recorders) used for data collection; if/how the instrument(s) changed over the course of the study	N/A
**Units of study** - Number and relevant characteristics of participants, documents, or events included in the study; level of participation (could be reported in results)	7; table 4
**Data processing** - Methods for processing data prior to and during analysis, including transcription, data entry, data management and security, verification of data integrity, data coding, and anonymization/de-identification of excerpts	5,6,7
**Data analysis** - Process by which inferences, themes, etc., were identified and developed, including the researchers involved in data analysis; usually references a specific paradigm or approach; rationale**	5,6
**Techniques to enhance trustworthiness** - Techniques to enhance trustworthiness and credibility of data analysis (e.g., member checking, audit trail, triangulation); rationale**	6 first paragraph

Results/findings

**Synthesis and interpretation** - Main findings (e.g., interpretations, inferences, and themes); might include development of a theory or model, or integration with prior research or theory	7,8 figure 2
**Links to empirical data** - Evidence (e.g., quotes, field notes, text excerpts, photographs) to substantiate analytic findings	Table 4,5,6,7

Discussion

**Integration with prior work, implications, transferability, and contribution(s) to the field** - Short summary of main findings; explanation of how findings and conclusions connect to, support, elaborate on, or challenge conclusions of earlier scholarship; discussion of scope of application/generalizability; identification of unique contribution(s) to scholarship in a discipline or field	8-11
**Limitations** - Trustworthiness and limitations of findings	3

Other

**Conflicts of interest** - Potential sources of influence or perceived influence on study conduct and conclusions; how these were managed	14
**Funding** - Sources of funding and other support; role of funders in data collection, interpretation, and reporting	14

*The authors created the SRQR by searching the literature to identify guidelines, reporting standards, and critical appraisal criteria for qualitative research; reviewing the reference lists of retrieved sources; and contacting experts to gain feedback. The SRQR aims to improve the transparency of all aspects of qualitative research by providing clear standards for reporting qualitative research.

For peer review only

For peer review only - http://bmjopen.bmj.com/site/about/guidelines.xhtml

**The rationale should briefly discuss the justification for choosing that theory, approach, method, or technique rather than other options available, the assumptions and limitations implicit in those choices, and how those choices influence study conclusions and transferability. As appropriate, the rationale for several items might be discussed together.

Reference:

O'Brien BC, Harris IB, Beckman TJ, Reed DA, Cook DA. **Standards for reporting qualitative research: a synthesis of recommendations.** *Academic Medicine*, Vol. 89, No. 9 / Sept 2014 DOI: 10.1097/ACM.0000000000000388

For peer review only

For peer review only - http://bmjopen.bmj.com/site/about/guidelines.xhtml
